# Heterozygous COL4A3/COL4A4 variants: diagnostic trends and clinical kidney outcomes

**DOI:** 10.1093/ckj/sfag301

**Published:** 2026-09-02

**Authors:** Yu Tanaka, Tomoko Horinouchi, Shuhei Aoyama, Yuka Kimura, Yuta Inoki, Yuta Ichikawa, Chika Ueda, Hideaki Kitakado, Atsushi Kondo, Nana Sakakibara, China Nagano, Tomohiko Yamamura, Shingo Ishimori, Kandai Nozu

**Affiliations:** Department of Pediatrics, Kobe University Graduate School of Medicine, Hyogo, Japan; Department of Pediatrics, Kobe University Graduate School of Medicine, Hyogo, Japan; Department of Pediatrics, Kobe University Graduate School of Medicine, Hyogo, Japan; Department of Pediatrics, Kobe University Graduate School of Medicine, Hyogo, Japan; Department of Pediatrics, Kobe University Graduate School of Medicine, Hyogo, Japan; Department of Pediatrics, Kobe University Graduate School of Medicine, Hyogo, Japan; Department of Pediatrics, Kobe University Graduate School of Medicine, Hyogo, Japan; Department of Pediatrics, Kobe University Graduate School of Medicine, Hyogo, Japan; Department of Pediatrics, Kobe University Graduate School of Medicine, Hyogo, Japan; Department of Pediatrics, Kobe University Graduate School of Medicine, Hyogo, Japan; Department of Pediatrics, Kobe University Graduate School of Medicine, Hyogo, Japan; Department of Pediatrics, Kobe University Graduate School of Medicine, Hyogo, Japan; Department of Pediatrics, Kobe University Graduate School of Medicine, Hyogo, Japan; Department of Pediatrics, Kobe University Graduate School of Medicine, Hyogo, Japan

**Keywords:** haematuria, heterozygous *COL4A3* or *COL4A4* variants, kidney failure, next-generation sequencing, proteinuria

## Abstract

**Background:**

Heterozygous pathogenic variants in *COL4A3* or *COL4A4* are relatively common and associated with a broad clinical spectrum. However, nomenclature and risk stratification remain unsettled. We aimed to investigate temporal diagnostic trends and clinical features of haematuria-positive individuals with heterozygous *COL4A3/COL4A4* variants in a genetic testing cohort.

**Methods:**

This retrospective cohort study involved 992 families genetically diagnosed with Alport syndrome at a single centre in Japan between 2006 and 2023. We identified 265 families comprising 299 haematuria-positive individuals with heterozygous pathogenic or likely pathogenic *COL4A3*/*COL4A4* variants. For kidney failure (KF) analysis, 627 individuals, including affected relatives, were evaluated.

**Results:**

At the family level, the proportion of the study group increased from 8.4% of genetically diagnosed Alport syndrome cases during the Sanger sequencing era (2006–2015) to 26.6% during early next-generation sequencing implementation (2015–2019), and 39.1% in the recent period (2020–2023). At genetic diagnosis, 75.3% of individuals had proteinuria and 4.7% had KF. Kaplan–Meier analysis showed that the median ages at first detection of haematuria and proteinuria, and at onset of KF, were 10, 26, and 72 years, respectively.

**Conclusions:**

Individuals with haematuria and heterozygous *COL4A3/COL4A4* variants accounted for an increasing proportion of genetically diagnosed cases of Alport syndrome. Although this referred cohort does not represent all individuals with heterozygous *COL4A3/COL4A4* variants, our findings delineate the natural history of haematuria-positive individuals with these variants and may support early diagnosis and risk stratification aimed at preventing progression to KF.

## Graphical Abstract

**Graphical Abstract:**
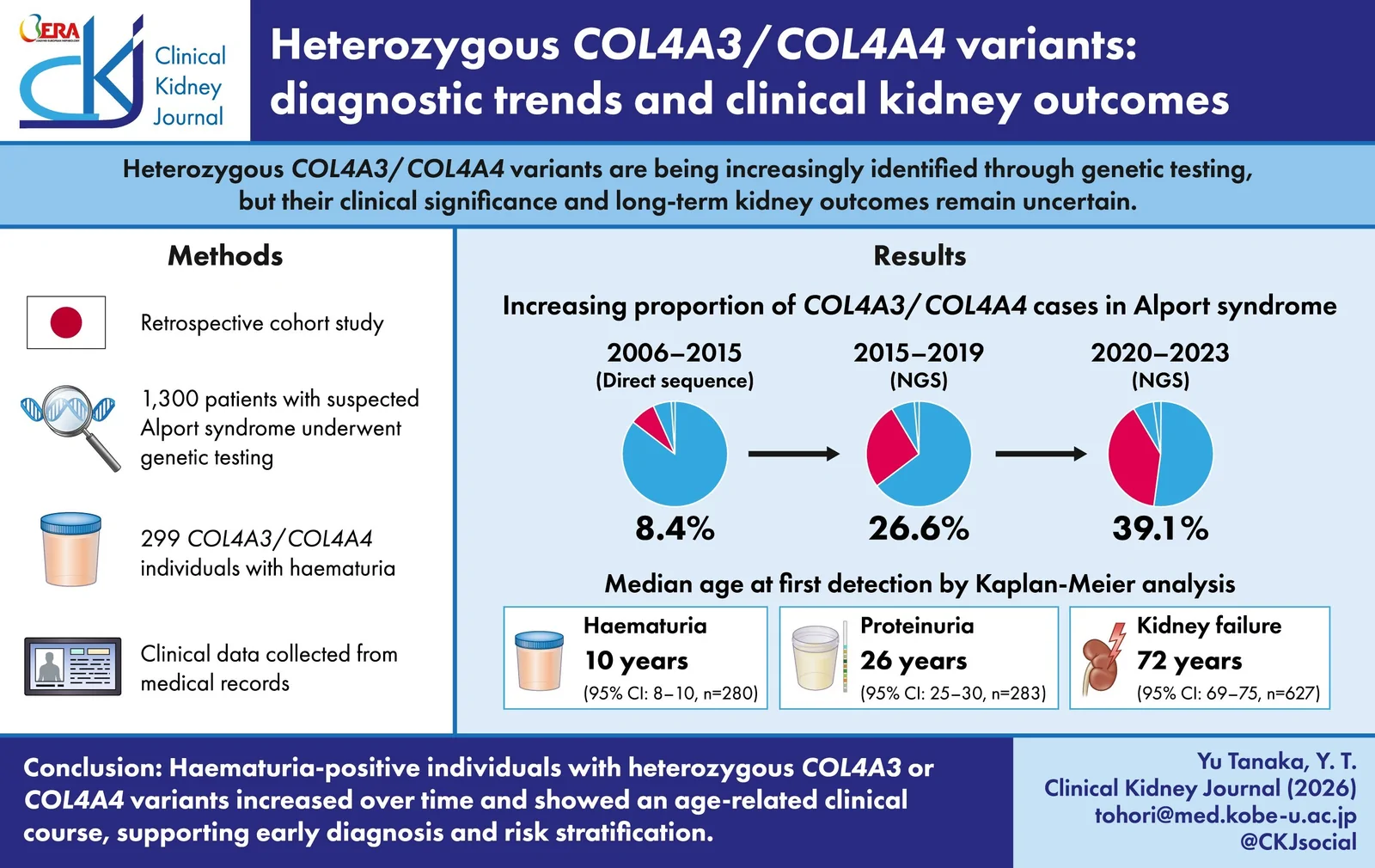

KEY LEARNING POINTS **What was known:**

- Heterozygous pathogenic variants in *COL4A3* or *COL4A4* are relatively common and are associated with a broad clinical spectrum from asymptomatic haematuria to kidney failure.
- Widespread use of next-generation sequencing increased the recognition of individuals with heterozygous *COL4A3/COL4A4* variants in clinical practice.
- However, terminology, clinical classification, and long-term risk stratification remain unsettled.

**This study adds:**

- In a Japanese genetic testing cohort, haematuria-positive individuals with heterozygous *COL4A3/COL4A4* variants accounted for an increasing proportion of genetically diagnosed Alport syndrome cases over time.
- Their proportion increased from 8.4% in the Sanger sequencing era to 26.6% during early next-generation sequencing implementation and 39.1% in the recent period.
- In this group, haematuria was first detected at a median age of 10 years, proteinuria at 26 years, and kidney failure at 72 years.

**Potential impact:**

- This referred cohort included individuals suspected of Alport syndrome and does not represent the full spectrum or penetrance among all individuals with heterozygous *COL4A3/COL4A4* variants; however, these variants remain an important differential diagnosis in asymptomatic haematuria.
- Clarifying the age-related natural history of haematuria, proteinuria, and kidney failure may improve risk stratification and inform follow-up strategies.
- Earlier genetic diagnosis may facilitate counselling, family screening, long-term monitoring, and timely initiation of renin–angiotensin system inhibition after the onset of proteinuria.

## INTRODUCTION

Alport syndrome (AS) is a progressive hereditary kidney disease caused by abnormalities in the type IV collagen α3, α4, and α5 chains and may be accompanied by sensorineural hearing loss and ocular abnormalities [1, 2]. Major genetic forms include X-linked AS (XLAS) due to pathogenic *COL4A5* variants, autosomal recessive AS (ARAS) due to biallelic *COL4A3* or *COL4A4* variants, and digenic AS involving variants in more than one *COL4A3–COL4A5* gene. Individuals with heterozygous *COL4A3/COL4A4* variants have been described using terms, such as ‘thin basement membrane nephropathy’, ‘benign familial haematuria’, and ‘autosomal dominant AS’; however, terminology remains controversial, and recent guidelines use ‘*COL4A3* and *COL4A4* heterozygotes’ [3, 4]. An international proposal has recently advocated the term ‘Alport Spectrum’ to better reflect the condition’s clinical heterogeneity [5].

Historically, AS was estimated to comprise approximately 80% XLAS, 15% ARAS, and 5% autosomal dominant AS [1]. However, with the widespread use of next-generation sequencing (NGS), individuals with heterozygous *COL4A3/COL4A4* variants are increasingly identified [6, 7]. Although relatively common in the general population, with a prevalence of 0.94% in the GnomAD database [8], the presence of a heterozygous *COL4A3/COL4A4* variant alone does not necessarily explain the kidney phenotype. Nevertheless, such variants have been associated with thin basement membrane nephropathy, kidney cysts, IgA nephropathy, and chronic kidney disease (CKD) [9–13]. Moreover, for individuals with heterozygous *COL4A3*/*COL4A4* variants, regular screening for microhaematuria and microalbuminuria (using UACR) at 1–2-year intervals, along with annual blood pressure monitoring, is recommended [3]. Although population- or family-based longitudinal studies are needed to clarify penetrance and natural history, these remain difficult because genetic testing is not routinely performed in asymptomatic individuals [14].

Given the evolving nomenclature and these clinical limitations, we focused on haematuria-positive individuals with heterozygous *COL4A3/COL4A4* variants identified in a clinically referred genetic testing cohort. We aimed to characterize their clinical features and temporal diagnostic trends among genetically confirmed AS cases, thereby supporting earlier recognition and appropriate long-term management.

## MATERIALS AND METHODS

### Study design and population

This retrospective cohort study included 1300 individuals with clinically suspected AS referred to our institution from across Japan for genetic testing between January 2006 and June 2023. No uniform referral criteria were applied, and referral was based on the clinical judgement of the referring physicians. For the present analysis, we identified haematuria-positive individuals with heterozygous *COL4A3/COL4A4* variants. Haematuria was defined as ≥5 red blood cells per high-power field on urinary sediment examination, confirmed on at least two occasions over ≥3 months [15]. This study group was defined for analytical purposes and was not intended to establish a universal disease definition or exclude asymptomatic individuals with heterozygous *COL4A3/COL4A4* variants from clinical surveillance.

### Clinical data collection

We retrospectively reviewed medical records during genetic testing to extract relevant clinical information, including age, sex, family history (FH), kidney function, and urinalysis findings. Serum creatinine-based estimated glomerular filtration rate (Cr-eGFR) was calculated using the Japanese-specific formulas, with separate equations applied to paediatric and adult patients based on age [16, 17]. Kidney failure (KF) was defined as a decline in Cr-eGFR to <10 ml/min/1.73 m^2^ or the initiation of kidney replacement therapy (haemodialysis, peritoneal dialysis, or kidney transplantation).

### Survival analyses

Kaplan–Meier analysis was used to estimate the age at first haematuria and proteinuria detection and the age at KF onset in genetically diagnosed haematuria-positive individuals with heterozygous *COL4A3/COL4A4* variants. Furthermore, KF onset analyses were performed for probands and affected family members. Affected family members were defined as individuals with haematuria who shared an autosomal dominant inheritance pattern with the index patient. To clarify long-term outcomes, second-degree relatives were also included only when first-degree relatives were affected and available for analysis. KF analyses, including affected relatives, were considered exploratory because multiple members from the same family were included.

### Genetic analysis

Genomic DNA was isolated from peripheral blood leukocytes using the Quick Gene Mini 80 System or QuickGene-Auto 12S (Kurabo Industries Ltd, Tokyo, Japan), following the manufacturer’s instructions. Mutational analyses of *COL4A3, COL4A4*, and *COL4A5* were performed using Sanger sequencing between January 2006 and November 2015. During this period, analyses were limited to these three genes: *COL4A5* was analysed first, while *COL4A3* and/or *COL4A4* were subsequently analysed when no pathogenic *COL4A5* variant was identified. Targeted panel sequencing was performed between November 2015 and June 2023 using a custom panel designed for genes involved in inherited kidney diseases, including *COL4A3, COL4A4, COL4A5*, and other kidney disease-associated genes (Supplementary Table S1). After the introduction of NGS, *COL4A3–COL4A5* and other inherited kidney disease-associated genes were analysed simultaneously. Sanger sequencing, targeted panel sequencing, multiplex ligation-dependent probe amplification (MLPA), polymerase chain reaction analysis of mRNA, and *in vitro* splicing assays using minigene constructs were performed as previously described [18]. MLPA was performed when copy-number variants (CNVs) were suspected, but systematic CNV screening was not performed.

### Variant categorization and evaluation

Pathogenic variants were determined according to the American College of Medical Genetics and Genomics criteria modified for AS [19], with ‘Pathogenic’ or ‘Likely Pathogenic’ designations considered disease-causing [20, 21]. Throughout this manuscript, unless otherwise specified, ‘variant’ denotes a pathogenic or likely pathogenic variant. Allele frequencies were referenced from the Genome Aggregation Database (gnomAD) to assess the prevalence of variants in the general population. *In silico* analyses were conducted using SIFT (http://sift.bii.a-star.edu.sg), PolyPhen2 (http://genetics.bwh.harvard.edu/pph2/), Mutation Taster (https://www.mutationtaster.org/), and CADD (https://cadd.gs.washington.edu/). Each variant was examined in the Human Gene Mutation Database ( https://www.qiagen.com/de-jp/product-categories/discovery-and-translational-research/next-generation) to determine whether a heterozygous mutation in that variant could cause urinary abnormalities. Truncating variants comprised nonsense and out-of-frame insertions/deletions, whereas non-truncating variants comprised missense variants and in-frame insertions/deletions. Canonical and non-canonical splice site variants were excluded from the truncating versus non-truncating comparison because their *in vivo* effects were unclear.

### Ethical considerations

All procedures were approved by the Institutional Review Board of Kobe University School of Medicine (IRB approval number: 301), and conducted following the principles of the Declaration of Helsinki and the ethical guidelines of the Japanese Ministry of Health, Labour, and Welfare. Informed consent was obtained from all patients or their parents.

### Statistical analysis

All statistical analyses were performed using EZR (Saitama Medical Center, Jichi Medical University, Saitama, Japan) [22], a modified version of R Commander (The R Foundation for Statistical Computing, Vienna, Austria) with additional statistical functions. Statistical significance was set at *P* < 0.05.

## RESULTS

### Distribution of haematuria-positive individuals with heterozygous COL4A3/COL4A4 variants

All analyses were performed at the family level. In our overall cohort of families with AS (*n* = 992 families), 65.1% (*n* = 646/992), 26.7% (*n* = 265/992), 6.6% (*n* = 65/992), and 1.6% (*n* = 16/992) were classified as XLAS, haematuria-positive individuals with heterozygous *COL4A3/COL4A4* variants, ARAS, and digenic AS, respectively (Fig. 1). The distribution of these genetic categories was evaluated across three periods: the era when direct sequencing was predominantly used (2006–2015; *n* = 251), the early phase of NGS implementation (2015–2019; *n* = 368), and the most recent period (2020–2023; *n* = 373). The proportion of haematuria-positive individuals with heterozygous *COL4A3/COL4A4* variants among families with AS during these periods was 8.4% (*n* = 21/251), 26.6% (*n* = 98/368), and 39.1% (*n* = 146/373), respectively (Fig. 2).

**Figure 1:**
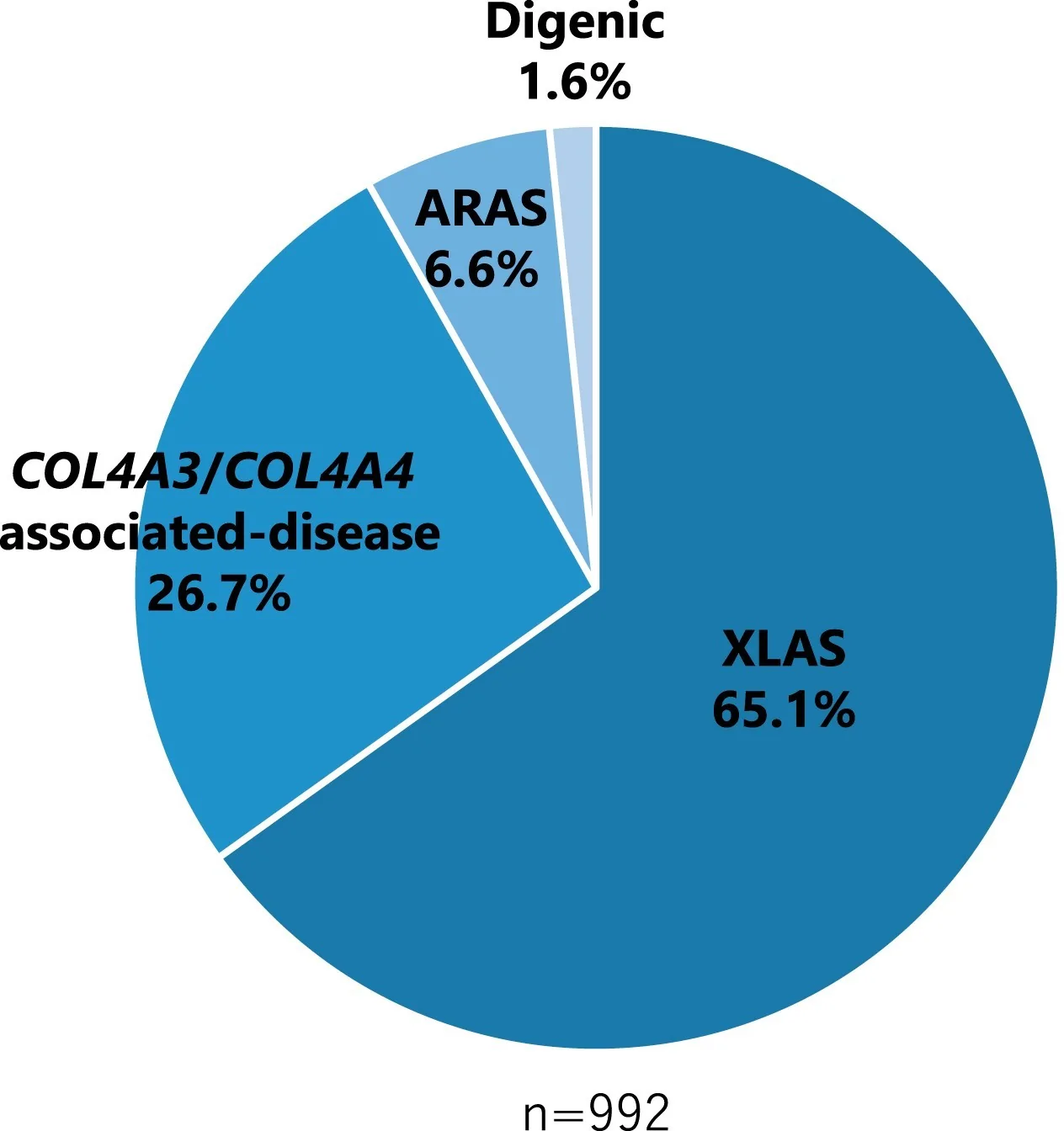
Distribution of genetic subtypes in AS and *COL4A3*/*COL4A4* associated-disease. A pie chart showing the proportions of genetic diagnoses among 992 families with genetically confirmed AS and *COL4A3/COL4A4* associated-disease. XLAS was the most common (65.1%), followed by *COL4A3*/*COL4A4* associated-disease (26.7%), ARAS (6.6%), and digenic inheritance (1.6%).

**Figure 2:**
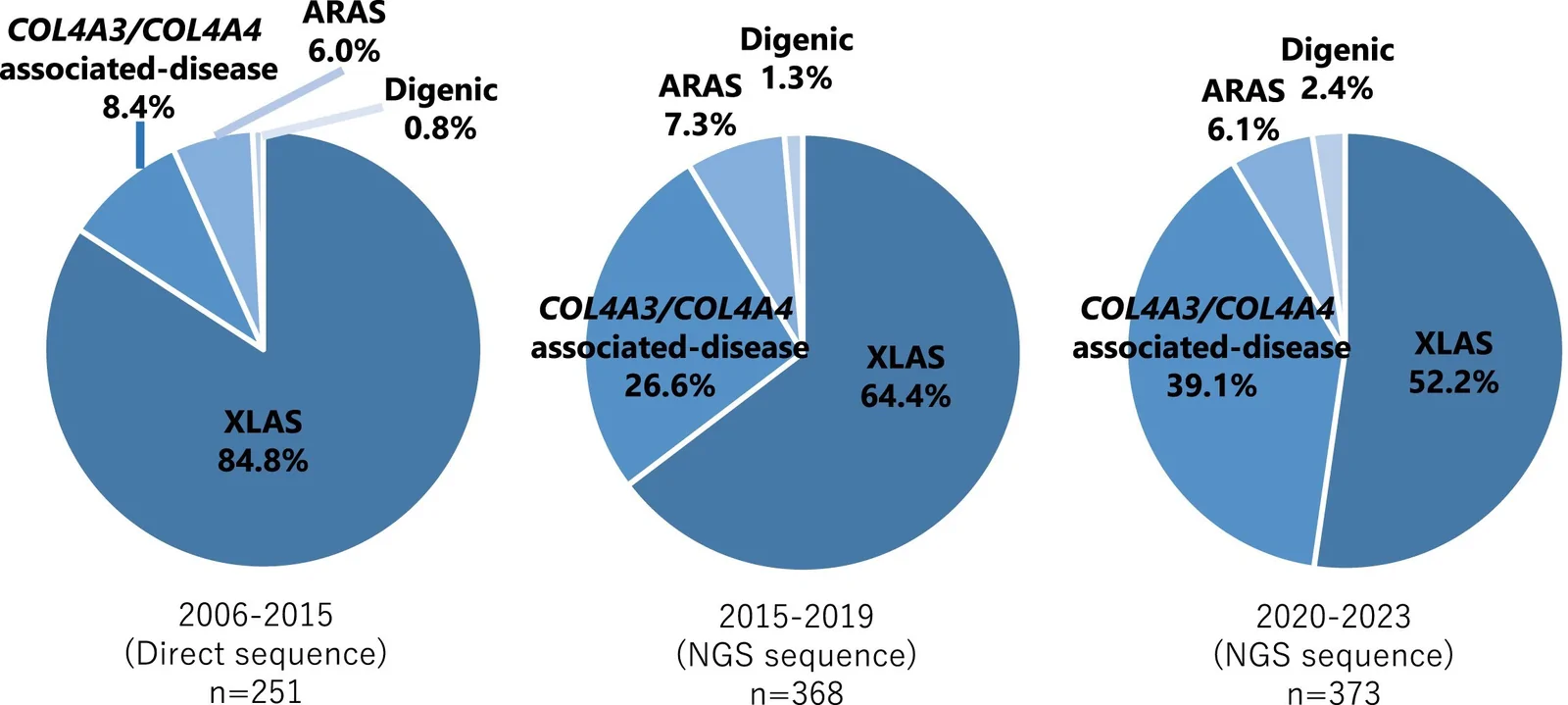
Temporal changes in the distribution of genetic subtypes in AS and *COL4A3*/*COL4A4* associated-disease. Pie charts illustrating the proportions of genetic subtypes diagnosed during three different periods: 2006–2015 (direct sequencing, *n* = 251), 2015–2019 (NGS, *n* = 368), and 2020–2023 (NGS, *n* = 373). The increasing proportion of *COL4A3*/*COL4A4* associated-disease and digenic cases over time reflects improved detection through comprehensive genetic analysis.

### Overview of haematuria-positive individuals with heterozygous COL4A3/COL4A4 variants

Three denominators were used according to the analysis: 265 families, 299 genetically diagnosed individuals, and 627 individuals, including affected family members for KF analysis. Overall, 265 families, comprising 299 haematuria-positive individuals with heterozygous *COL4A3/COL4A4* variants, were identified (Supplementary Table S2). For the 299 genetically diagnosed individuals, the median age at genetic diagnosis was 42 years (interquartile range [IQR]: 26–52), and 64.2% (*n* = 192/299) were female (Table 1).

**Table 1:** Clinical characteristics at genetic diagnosis.

Clinical characteristics		
Median age (year [IQR])	42	(26‒52)
Female sex (% [*n*])	64.2	(192/299)
Family history of haematuria or KF (%[families])	83.0	(220/265)
Genetic testing before kidney biopsy (%[families with available data])	17.0	(44/259)
Haematuria (% [*n*])	100	(299/299)
Proteinuria (% [*n*])	75.3	(225/299)
Kidney failure (% [*n*])	4.7	(14/299)

IQR: interquartile range, KF: kidney failure.

Among 265 families, 83.0% (*n* = 220/265) had a FH of haematuria or KF at the time of genetic testing. Among the 220 families with a positive FH, 126 had a FH of haematuria without KF, and 94 had a FH of KF. Among the 94 families with a FH of KF, 69 had a first-degree vertical relative, defined as a parent or child, with KF. In families with available data, genetic testing was performed before kidney biopsy in 17.0% (*n* = 44/259). This genetic-first approach increased over time, from 4.8% in 2006–2015 to 21.7% in 2020–2023 (Supplementary Table S3).

### Kidney phenotypes

By definition, documented haematuria was present in all genetically diagnosed individuals included in this group (*n* = 299/299) , whereas 75.3% (*n* = 225/299) had proteinuria at diagnosis. KF was observed in 4.7% (*n* = 14/299) of patients at the time of genetic testing (Table 1). Kaplan–Meier analysis revealed median ages at first detection of haematuria and proteinuria of 10 years (95% confidence interval [CI]: 8–10, *n* = 280) and 26 years (95% CI: 25–30, *n* = 283), respectively. The median age at KF was 72 years (95% CI: 69–75; *n* = 627) when both probands and affected family members were included (Figs 3 and 4). Detailed clinical data for the 14 genetically diagnosed individuals with KF are shown in Supplementary Table S4. Among the nine individuals with available data, seven had received renin–angiotensin system (RAS) inhibitor therapy. Comorbidities included obesity (*n* = 6), dyslipidaemia (*n* = 6), hyperuricaemia (*n* = 4), and hypertension (*n* = 3). Diabetes mellitus and kidney diseases other than AS were not documented.

**Figure 3:**
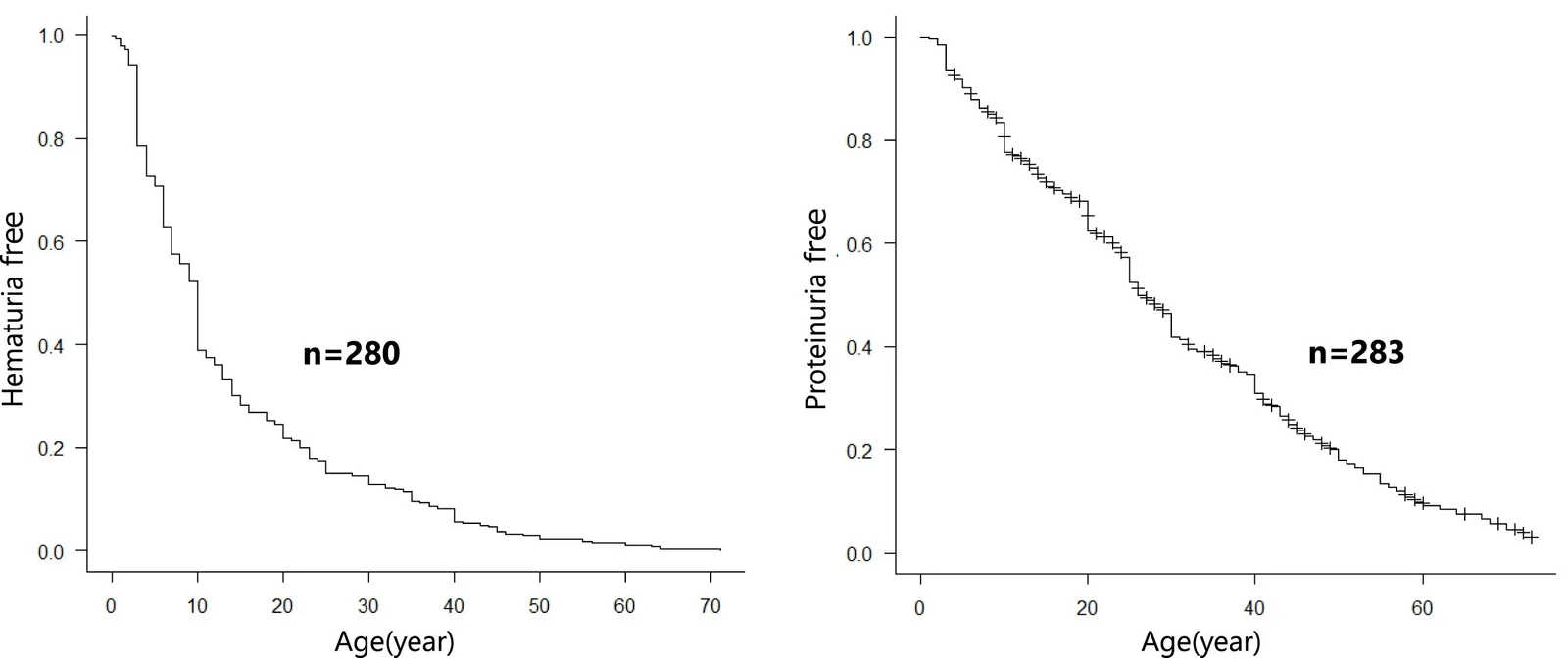
Kaplan–Meier curves for age at first detection of haematuria and proteinuria in haematuria-positive individuals with heterozygous *COL4A3*/*COL4A4* variants. Kaplan–Meier survival estimates for age at first detection of haematuria (left, *n* = 280) and proteinuria (right, *n* = 283) among patients with genetically diagnosed with *COL4A3*/*COL4A4* associated-disease.

**Figure 4:**
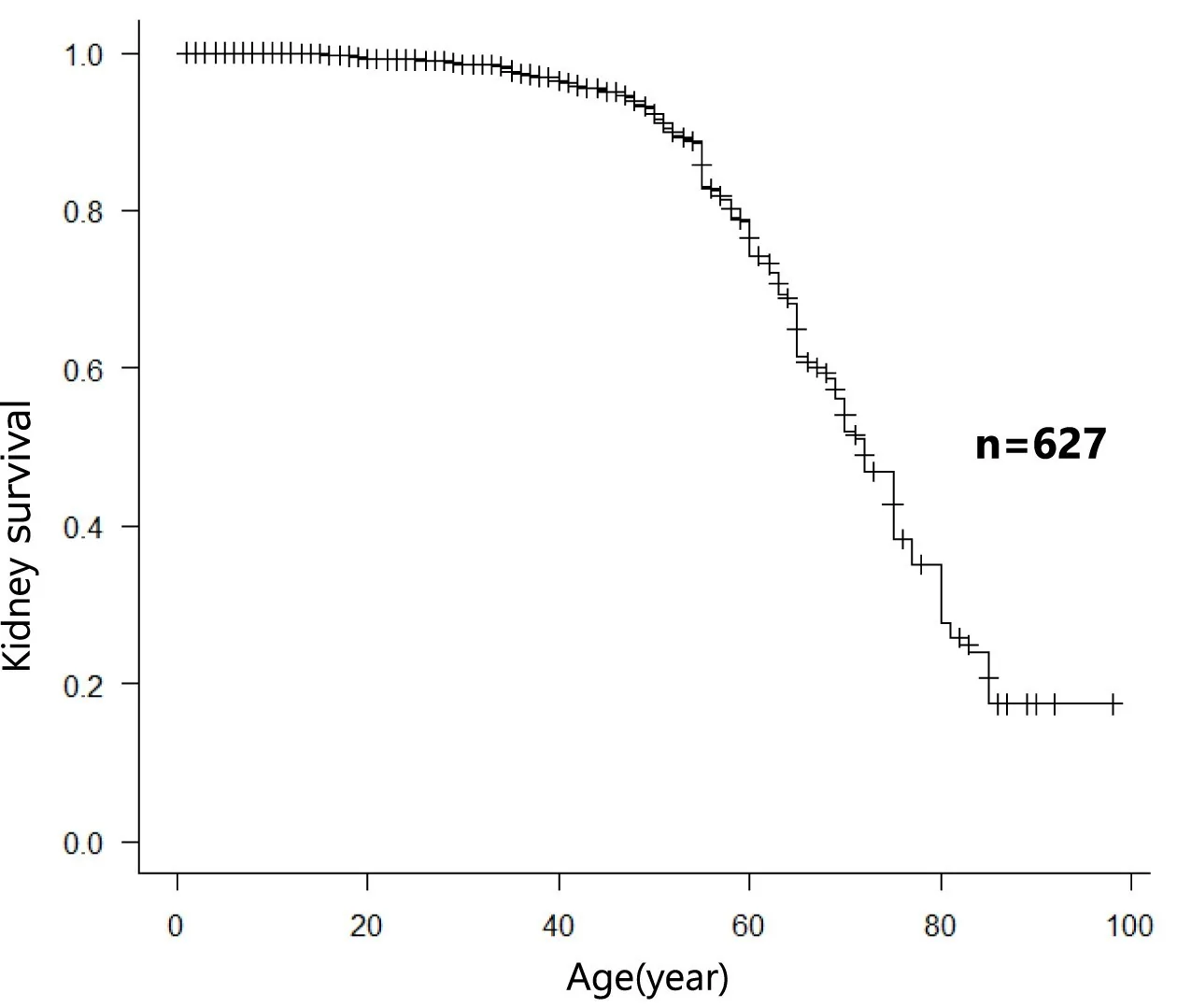
Kaplan–Meier curve for kidney survival, including individuals and affected family members. Kaplan–Meier analysis of kidney survival showing the age at onset of KF in a combined cohort of individuals and affected relatives with *COL4A3*/*COL4A4* associated-disease (*n* = 627).

### Genotypes

Pathogenic variants were identified in the *COL4A3* gene in 42.3% of families (*n* = 112/265) and in *COL4A4* in 57.7% of families (*n* = 153/265). By variant type, 74.7% (*n* = 198/265) were missense variants, 11.3% (*n* = 30/265) were splice site variants, 8.0% (*n* = 21/265) were insertions/deletions (5 in-frame and 16 out-of-frame), and 6.0% (*n* = 16/265) were nonsense variants.

### Genotype–phenotype correlations

To investigate genotype–phenotype correlations, we compared the age at KF among haematuria-positive individuals with heterozygous *COL4A3/COL4A4* variants and affected family members between individuals harbouring truncating and non-truncating variants. No significant differences were observed in the median age at KF (75 vs. 70 years, *P* = 0.83, *n* = 568) (Fig. 5).

**Figure 5:**
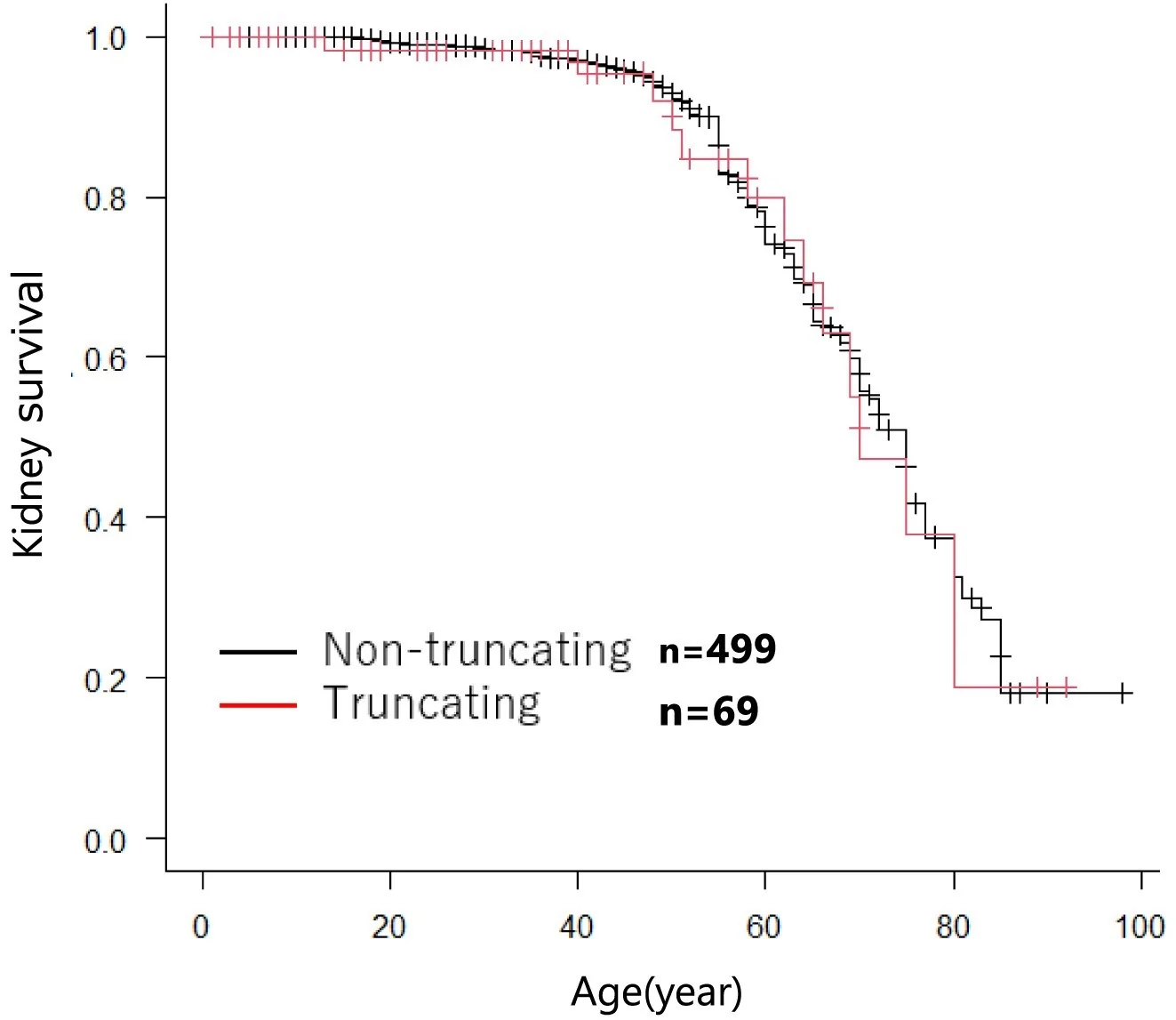
Kaplan–Meier curves for kidney survival by variant type in *COL4A3*/*COL4A4*. Kaplan–Meier analysis showing kidney survival stratified by truncating (*n* = 69) and non-truncating (*n* = 499) variants in haematuria-positive individuals with heterozygous *COL4A3*/*COL4A4* variants.

### Individuals with eGFR <60 ml/min/1.73 m² in the absence of proteinuria at genetic diagnosis

Despite no evidence of proteinuria at diagnosis, 5.0% (*n* = 15/299) of individuals had an eGFR <60 ml/min/1.73 m². Their median age at diagnosis was 51 years. Kidney biopsy with electron microscopy was performed in 10 individuals, all of whom showed glomerular basement membrane thinning. Furthermore, in three individuals, a basket-weave change, characteristic of XLAS, was also observed. Genetically, 11 families (14 individuals) had missense variants, whereas one family (one individual) exhibited a splice site variant. The median age at first detection of haematuria was 16 years. The median interval between first detection of haematuria and genetic diagnosis was 32 years. Based on longitudinal clinical records before genetic diagnosis, five individuals had transient proteinuria that subsequently resolved, whereas the remaining 10 had no documented proteinuria during clinical follow-up before genetic diagnosis. The median Cr-eGFR at diagnosis was 48 ml/min/1.73 m². In total, 33.3% (*n* = 5/15) of these individuals were on RAS inhibitors. Three individuals had a history of gross haematuria, three had obesity, and three had hypertension, one of whom had hyperuricaemia; another had hypothyroidism. No individuals had a documented history of kidney disease other than AS in the available clinical records (Supplementary Table S5).

## DISCUSSION

In this retrospective cohort study, we focused on haematuria-positive individuals with heterozygous *COL4A3/COL4A4* variants in a clinically referred genetic testing cohort for suspected AS. We elucidated the temporal trends in the proportion of this group among all genetically diagnosed AS cases and characterized its clinical features. In the most recent NGS era, this group accounted for 39.1% of cases, and the median ages at first detection of haematuria and proteinuria and at KF onset were 10, 26, and 72 years, respectively.

Although what has traditionally been termed autosomal dominant AS has been estimated to account for ~5% of clinically diagnosed AS cases [1], NGS-based genetic testing, population and familial CKD screening have increased recognition of pathogenic *COL4A3/COL4A4* variants and their contribution to CKD [8, 12, 23, 24]. In this study, the proportion of individuals with heterozygous *COL4A3/COL4A4* variants increased markedly after NGS implementation, accounting for 26.6% of cases in 2015–2019 and 39.1% in 2020–2023. The rate of genetic-first diagnoses increased from 4.8% during the Sanger era to 21.7% in the most recent period. These findings suggest that the observed increase likely reflects improved recognition of heterozygous *COL4A3/COL4A4* variant-associated disease and broader use of genetic testing, rather than a true increase in disease incidence.

In our cohort, KF was observed at the time of genetic testing in 4.7% of genetically diagnosed individuals, which appeared lower than the 15%–25% reported in previous studies [25, 26], and closer to the 3% estimated in other reports [27]. Among these 14 individuals, obesity, dyslipidaemia, hyperuricaemia, and hypertension were documented in some cases, whereas diabetes mellitus and kidney disease other than AS were not identified in the available clinical records; however, incomplete data on RAS inhibitor timing, intensity, and duration precluded evaluation of treatment effects. The median age of KF was 72 years when all 627 individuals and affected family members were included. A previous analysis of 82 families (252 individuals) reported a median age of KF onset of 58 years [25], whereas a systematic review involving 48 studies (777 individuals) indicated that 15% progressed to KF at an average age of 52.8 years [26]. Another investigation of 403 individuals harbouring pathogenic *COL4A3* variants in an unselected health system-based cohort reported a median age at KF onset of 58.3 years [28]. Some of these studies included individuals without haematuria, suggesting that patient backgrounds might differ and complicate direct comparisons. Because this was a clinically ascertained referral cohort enriched for individuals with suspected AS, direct comparison with age-specific population-level KF risks was limited and should be interpreted with caution. Additionally, as our KF analysis included both individuals and affected family members, the results may have been influenced by family clustering and non-independent observations. These differences in findings may be attributable to variations in cohort characteristics across studies.

We elucidated the clinical course of haematuria-positive individuals with heterozygous *COL4A3*/*COL4A4* variants by determining the age of KF and analysing the age at first detection of haematuria and proteinuria. Traditionally, AS has been characterized by asymptomatic haematuria from early childhood, particularly in XLAS and ARAS [14]. However, in individuals with heterozygous *COL4A3*/*COL4A4* variants, haematuria often first appears in adolescence or later, which can delay the diagnosis due to similarities with other chronic glomerulopathies. Moreover, a latency of >10 years may separate the onset of haematuria from proteinuria development, creating a substantial risk for misdiagnosis as ‘benign haematuria’. Therefore, even if haematuria alone persists for many years, clinicians should consider heterozygous *COL4A3*/*COL4A4* variant-associated-disease and ensure continuous follow-up evaluations.

Although multiple studies have investigated genotype–phenotype correlations in heterozygous *COL4A3*/*COL4A4* variant-associated-disease, no definitive consensus has been reached. In a retrospective analysis of 16 families (72 individuals) from our institution, no significant difference was observed in the age at KF onset between patients with missense and non-missense variants [29]. Similarly, a cohort study of 82 families (252 individuals) reported no differences in KF between truncating and non-truncating variants or among other variant types [25]. Here, no significant differences were observed in the KF onset age among 568 individuals, regardless of truncating or non-truncating variants. In contrast, a systematic review of 258 families (777 individuals) reported that non-missense variants were associated with earlier progression to KF than missense variants (mean age, 47.1 vs. 55.2 years) [26]. Furthermore, a recent study of 89 individuals with *COL4A3/COL4A4* variants reported that monoallelic non-truncating variants were associated with a more severe phenotype than monoallelic truncating variants, suggesting a possible dominant-negative effect [30]. Several factors may explain these inconsistencies. First, patient selection criteria for heterozygous *COL4A3*/*COL4A4* variant-associated-disease likely vary among studies. Second, the number of individuals with truncating variants in our cohort was limited, and the genotype–phenotype analysis may have been underpowered to detect modest differences. Third, as KF typically occurs at an older age in this group, non-genetic modifiers—such as lifestyle factors and comorbidities—may exert a stronger influence, potentially masking the effects of specific genotypes.

Although *COL4A3/COL4A4* variant-associated-disease does not progress to CKD without proteinuria [25], our findings indicate otherwise. In our study, 15 individuals (5.0% of the cohort) exhibited an eGFR <60 ml/min/1.73 m^2^ despite having no proteinuria on record. Of these, five were on RAS inhibitors, yet four had never developed proteinuria. Three had hypertension, which was well controlled with antihypertensive drugs, and no other apparent comorbidities that could induce kidney dysfunction were identified. Furthermore, all 10 individuals who underwent electron microscopy showed glomerular basement membrane changes characteristic of AS, and three families (six patients) demonstrated a similar clinical course (Supplementary Fig. S1). Since the implementation of NGS in 2015, which simultaneously screens over 130 kidney disease-associated genes, no pathogenic variants other than *COL4A3* and *COL4A4* have been detected. Therefore, *COL4A3/COL4A4* variant-associated-disease can lead to KF, not only through the typical AS disease course, but also as a consequence of CKD risk associated with *COL4A3* or *COL4A4* variants, resulting in kidney function decline even without proteinuria. However, because kidney impairment in *COL4A3/COL4A4* variant-associated-disease generally progresses slowly, the involvement of ageing, environmental factors, and yet-undetermined risk factors cannot be excluded. Further case accumulation and long-term follow-up studies are warranted to more precisely elucidate the underlying disease mechanisms.

This study has some limitations. First, this was a single-centre retrospective study of individuals referred for genetic testing because AS or *COL4A3/COL4A4* variant-associated-disease was clinically suspected. Therefore, the cohort may be biased towards individuals with more clinically apparent or severe kidney manifestations, characteristic biopsy findings, or a positive FH, and our findings cannot be used to estimate the penetrance or population-based prognosis of all individuals with heterozygous *COL4A3/COL4A4* variants. In addition, because this was an entirely Japanese cohort, differences in ancestry may limit the comparability of disease course with cohorts from other populations. Second, we could not definitively exclude ARAS, digenic AS, undetected deep intronic or structural variants, or other genetic or acquired kidney diseases, because only gene-panel NGS, not genome-wide NGS such as exome or genome sequencing, was performed. In addition, urinary red blood cell morphology was not consistently recorded across referring institutions, and a glomerular origin of haematuria could not be confirmed in all individuals. Third, the KF analysis included both individuals and affected family members and may therefore have been influenced by family clustering and non-independent observations. As cascade testing was not systematically performed, the familial *COL4A3/COL4A4* variant was not confirmed in all affected relatives included in the KF analysis, and we could not determine whether KF was solely attributable to this variant in every case. Fourth, the number of individuals with truncating variants was limited, and the genotype–phenotype analysis may have been underpowered to detect modest differences. In addition, canonical and non-canonical splice site variants were excluded from the truncating vs. non-truncating comparison, and systematic CNV screening was not performed; these factors may limit comparability with studies that classify canonical splice site variants or CNVs as truncating variants. Finally, although our findings suggest kidney function decline without documented proteinuria in some individuals, this observation should be interpreted cautiously because ageing, environmental factors, comorbidities, retrospective variability in proteinuria assessment, and unknown or superimposed kidney disorders could not be fully excluded. Larger multicentre cohorts and long-term prospective studies including both symptomatic and asymptomatic individuals with heterozygous *COL4A3/COL4A4* variants are needed to clarify the full clinical spectrum, penetrance, and mechanisms underlying kidney function decline in this disease.

In conclusion, this study elucidated the temporal trends in the proportion of haematuria-positive individuals with heterozygous *COL4A3/COL4A4* variants among all genetically diagnosed AS cases in a referred genetic testing cohort. We characterized the clinical features of this group, which often begins with haematuria detected during childhood or adolescence, progresses over more than a decade to proteinuria, and eventually leads to KF at an old age. Although the understanding and nomenclature of this disease are still evolving, the widespread use of genetic testing may increase the identification of individuals with heterozygous *COL4A3/COL4A4* variant-associated-disease.

## Supplementary Material

sfag301_Supplemental_File

## Data Availability

The data underlying this article will not be shared publicly due to the absence of participant consent for data sharing and institutional restrictions. Data may be made available to editors upon request for manuscript review.
