# Supplementary material for: Heterozygous COL4A3/COL4A4 variants: diagnostic trends and clinical kidney outcomes
Source: Clin Kidney J. 2026 Sep 2;19(10):sfag301. doi: 10.1093/ckj/sfag301 (PMC13627838; doi:10.1093/ckj/sfag301)
Supplement: Supplementary file 1 [file sfag301_supplemental_file.docx]

**Temporal trends in the diagnosis rates and clinical characteristics of haematuria-positive individuals with heterozygous *COL4A3* or *COL4A4* variants in a genetic testing cohort: A retrospective cohort study**

Yu Tanaka^1^, Tomoko Horinouchi^1*^, Shuhei Aoyama^1^, Yuka Kimura^1^, Yuta Inoki^1^, Yuta Ichikawa^1^, Chika Ueda^1^, Hideaki Kitakado^1^, Atsushi Kondo^1^, Nana Sakakibara^1^, China Nagano^1^, Tomohiko Yamamura^1^, Shingo Ishimori^1^, Kandai Nozu^1^

^1^ Department of Pediatrics, Kobe University Graduate School of Medicine, Hyogo, Japan

**Correspondence**:

Tomoko Horinouchi MD, PhD

Department of Pediatrics, Kobe University Graduate School of Medicine, 7‑5‑1 Kusunoki‑cho, Chuo‑ku, 650‑0017, Kobe, Japan

Tel: +81-78-382-6090; Fax: +81-78-382-6099; E-mail: [tohori@med.kobe-u.ac.jp](mailto:tohori@med.kobe-u.ac.jp)

ORCID: 0000-0003-1655-6030

**Supplementary Table S1:** Genes included in the next-generation sequencing (NGS) panel

**Supplementary Table S2:** Genetic variants identified in the study cohort

**Supplementary Table S3:** Clinical characteristics of individuals in the study cohort

**Supplementary Table S4:** Clinical characteristics of individuals with eGFR <60 mL/min/1.73 m² in the absence of proteinuria

**Supplementary Figure S1:** Pedigrees of individuals with eGFR <60 mL/min/1.73 m² in the absence of proteinuria

Family trees of six affected families (A1275, A568, and A1130 shown) in which at least one individual presented with an estimated glomerular filtration rate (eGFR) below 60 mL/min/1.73 m² despite the absence of overt proteinuria (UP−). OB: occult blood in urine; UP: urinary protein; Cr-eGFR: creatinine-based estimated glomerular filtration rate; KRT: kidney replacement therapy; d.: deceased.

**Alt text:** Pedigrees of affected families showing individuals with reduced eGFR despite no overt proteinuria and their familial segregation of *COL4A3* or *COL4A4* variants.

Supplementary Table S1

| *SLC12A1* | *SLC2A2* | *COQ8B (ADCK4)* | *TPRKB* |
| --- | --- | --- | --- |
| *KCNJ1* | *BCS1L* | *TTC21B* | *TP53RK* |
| *CLCNKB* | *GATM* | *NUP93* | *WDR4* |
| *BSND* | *HNF4A* | *NUP107* | *WDR35* |
| *CLCNKA* | *NDUFAF6* | *NUP205* | *WDR73* |
| *SLC12A3* | *CTNS* | *NUP85* | *PRDM15* |
| *CASR* | *NR3C2* | *NUP133* | *TRIM8* |
| *MAGED2* | *SCNN1A* | *NUP160* | *PODXL* |
| *CLDN10* | *SCNN1B* | *CRB2* | *TBC1D8B* |
| *CFTR* | *SCNN1G* | *CUBN* | *MAFB* |
| *CLCN5* | *KLHL3* | *EMP2* | *DAAM2* |
| *OCRL* | *CUL3* | *FAT1* | *EHHADH* |
| *EHD1* | *WNK1* | *KANK1* | *MYO1E* |
| *SLC26A3* | *WNK4* | *KANK2* | *LAGE3* |
| *KCNJ10* | *AQP2* | *KANK4* | *SLC34A1* |
| *CLDN16* | *AVPR2* | *PDSS2* | *ARHGDIA* |
| *CLDN19* | *AVP* | *PTPRO* | *OSGEP* |
| *FXYD2* | *COL4A3* | *XPO5* | *CA2* |
| *EGF* | *COL4A4* | *ACTN4* | *GLEPP1 (PTPRO)* |
| *TRPM6* | *COL4A5* | *ANLN* | *GON7* |
| *TRPM7* | *P3H2* | *ARHGAP24* | *ATP6V0A4* |
| *KCNA1* | *CD151* | *INF2* | *COQ6* |
| *CNNM2* | *GLA* | *LMX1B* | *LAMA5* |
| *HNF1B* | *UMOD* | *MYH9* | *ATP6V1B1* |
| *PCBD1* | *MUC1* | *PAX2* | *SLC4A4* |
| *ANK3* | *SEC61A1* | *TRPC6* | *ITGA3* |
| *CFH* | *REN* | *WT1* | *ITGB4* |
| *CFI* | *EYA1* | *MAGI2* | *GAPVD1* |
| *MCP (CD46)* | *SIX2* | *AVIL* | *ANKFY1* |
| *C3* | *CD2AP* | *TNS2* |  |
| *CFB* | *NPHS1* | *DLC1* |  |
| *DGKE* | *NPHS* | *CDK20* |  |
| *THBD* | *PLCE1（NPHS3）* | *ITSN1* |  |
| *CFHR1* | *SMARCAL1* | *ITSN2* |  |
| *ADAMTS13* | *LAMB2* | *KIRREL1* |  |
| *FN1* | *SCARB2* | *SGPL1* |  |
| *SLC4A1* | *COQ2* | *LMNA* |  |

Supplementary Table S2

| Patient | Family | Gene | Base | Amino | Report | gnomAD exomes  allele frequency | Insilico (CADD, Mutation Taster, Polyphen2, SIFT） | Clinvar  Clinical Significance | ACMG criteria |  |
| --- | --- | --- | --- | --- | --- | --- | --- | --- | --- | --- |
| 72 | 1 | *COL4A4* | c.2510G>C | p.G837A | Kamiyoshi(2016)Clin J Am Soc Nephrol | 0.00048518 | (25.3/D/PD/D) | Pathogenic, Likely pathogenic | Likely pathogenic (PM1, PM2, PP2, PP3, PP4) |  |
| 86 | 2 | *COL4A4* | c.2510G>C | p.G837A | Kamiyoshi(2016)Clin J Am Soc Nephrol | 0.00048518 | (25.3/D/PD/D) | Pathogenic, Likely pathogenic | Likely pathogenic (PM1, PM2, PP2, PP3, PP4) |  |
| 129 | 3 | *COL4A3* | c.2980+2T>A |  | Kamiyoshi(2016)Clin J Am Soc Nephrol | NA | NA | NA | Pathogenic (PVS1, PM2, PP4) |  |
| 161 | 4 | *COL4A3* | c.1855G>C | p.G619R |  | NA | (24.5/D/PD/D) | NA | Likely pathogenic (PM1, PM2, PP2, PP3) |  |
| 168 | 5 | *COL4A3* | c.2039G>A | p.G640R |  | 0 | (25.1/NA/PD/D) | NA | Likely pathogenic (PM1, PM2, PP1, PP2, PP3, PP4) |  |
| 175 | 6 | *COL4A3* | c.3499G>A | p.G1167R | Gao(2022)Front Genet | 2.2288×e-5 | (32/D/PD/D) | Pathogenic | Likely pathogenic (PM1, PM2, PP2, PP3, PP4） |  |
| 198 | 7 | *COL4A3* | c.1901G>A | p.G634E | Kamiyoshi(2016)Clin J Am Soc Nephrol | 0 | (24.1/D/PD/D) | NA | Likely pathogenic (PM1, PM2, PP1, PP2, PP3, PP4) |  |
| 153 | 9 | *COL4A4* | c.1733G>T | p.G578V | Oka(2014)Pediatr Nephrol | NA | (25.7/D/PD/D) | Pathogenic | Likely pathogenic (PM1, PM2, PP1, PP2, PP3, PP4) |  |
| 154 | 10 | *COL4A4* | c.2726G>A | p.G909E | Kamiyoshi(2016)Clin J Am Soc Nephrol | NA | (23.7/D/PD/D) | NA | Likely pathogenic (PM1, PM2, PP2, PP3, PP4) |  |
| 205 | 11 | *COL4A3* | c619G>A | p.G207R | Gao(2022)Front Genet | NA | (31/D/PD/D) | NA | Likely pathogenic (PM1, PM2, PP2, PP3, PP4) |  |
| 269 | 13 | *COL4A4* | c.4847T>G | p.L1616R | Imafuku(2017) Nephrology | 7.2692×e-5 | (28.9/D/PD/D) | NA | Likely pathogenic (PM1, PM2, PP2, PP3, PP4) |  |
| 274 | 14 | *COL4A4* | c.3687dupA | p.G230Rfs*23 | Horinouchi(2020)Kidney360 | 0 | NA | Pathogenic | Pathogenic (PVS1, PM2) |  |
| 280 | 15 | *COL4A4* | c.4847T>G | p.L1616R | Imafuku(2017) Nephrology | 7.2692×e-5 | (28.9/D/PD/D) | NA | Likely pathogenic (PM1, PM2, PP2, PP3, PP4) |  |
| 285 | 16 | *COL4A4* | c.2641delC | p.H881fs | Kamiyoshi(2016)Clin J Am Soc Nephrol | NA | NA | NA | Pathogenic (PVS1, PM2, PP4) |  |
| 291 | 17 | *COL4A4* | c.4064G>A | p.G1358E |  | NA | ('24.5/D/PD/D) | NA | Likely pathogenic (PM1, PM2, PP2, PP3) |  |
| 297 | 18 | *COL4A4* | c.2084G>A | p.G695D | Horinouchi(2020)Kidney360 | 2.7739×e-5 | (23.3/D/PD/D) | Likely pathogenic | Likely pathogenic (PM1, PM2, PP2, PP3, PP4) |  |
| 304 | 19 | *COL4A3* | c.2330G>A | p.G777D | Oka(2014)Pediatr Nephrol | NA | (29.4/D/PD/D) | NA | Likely pathogenic (PM1, PM2, PP2, PP3, PP4) |  |
| 305 | 20 | *COL4A3* | c.3464G>A | p.G1155D | Oka(2014)Pediatr Nephrol | 8.3195×e-5 | (27.2/D/PD/D) | Pathogenic | Likely pathogenic (PM1, PM2, PP2, PP3, PP4) |  |
| 320 | 21 | *COL4A3* | c.2863G>A | p.G955R | Imafuku(2017) Nephrology | 0.00011091 | (31/D/PD/D) | Likely pathogenic | Likely pathogenic (PM1, PM2, PP2, PP3, PP4) |  |
| 335 | 22 | *COL4A3* | c.3464G>A | p.G1155D | Oka(2014)Pediatr Nephrol | 8.3195×e-5 | (27.2/D/PD/D) | Pathogenic | Likely pathogenic (PM1, PM2, PP2, PP3, PP4) |  |
| 336 | 23 | *COL4A3* | c.4207G>A | p.G1403R | Imafuku(2017) Nephrology | 0.00027728 | (32/D/PD/D) | NA | Likely pathogenic (PM1, PM2, PP1, PP2, PP3, PP4) |  |
| 350 | 24 | *COL4A3* | c.899G>A | p.G300R | Mencarelli(2015) J Med Genet | 0 | (28.2/D/PD/D) | Conflicting | Likely pathogenic (PM1, PM2, PP1, PP2, PP3, PP4) |  |
| 351 | 25 | *COL4A3* | c.4793T>G | p.L1598R | Oka(2014)Pediatr Nephrol | 0.00075754 | (28.4/D/PD/D) | Conflicting | Likely pathogenic (PM1, PM2, PP1, PP2, PP3, PP4) |  |
| 363 | 26 | *COL4A3* | c.2981-2A>C |  |  | NA | NA | NA | Pathogenic (PVS1, PM2, PP4) |  |
| 367 | 27 | *COL4A3* | c.943G>A | p.G315S | Garcia-Aznar(2022) J Clin Med | NA | (26.2/D/PD/D) | NA | Likely pathogenic (PM1, PM2, PP2, PP3, PP4) |  |
| 369 | 28 | *COL4A3* | c.4928G>A | p.R1643K | Oka(2014)Pediatr Nephrol | 2.519×e-5 | (33/D/PD/D) | VUS | Likely pathogenic (PM1, PM2. PP2, PP3) |  |
| 376 | 29 | *COL4A3* | c.2972G>A | p.G991A |  | NA | (23.9/D/PD/D) | NA | Likely pathogenic (PM1, PM2, PP2, PP3) |  |
| 315 | 30 | *COL4A4* | c.4129C>T | p.R1377* | Boye(1998)Am J Hum Genet | 0 | NA | Pathogenic | Pathogenic (PVS1, PM2, PP1, PP4) |  |
| 319 | 31 | *COL4A4* | c.1057G>C | p.G353R | Imafuku(2017) Nephrology | NA | (26.0/D/PD/D) | NA | Likely pathogenic (PM1, PM2, PP2, PP3, PP4） |  |
| 330 | 32 | *COL4A4* | c.1862dupC | p.G622Rfs35 |  | 0 | NA | NA | Pathogenic (PVS1, PM2, PP4) |  |
| 331 | 33 | *COL4A4* | c.827G>C | p.G276A | Imafuku(2017) Nephrology | 0.00089806 | (25.5/D/PD/D) | Likely pathogenic | Pathogenic (PM1, PM2, PP3, PP4) |  |
| 332 | 34 | *COL4A4* | c.2510G>C | p.G837A | Kamiyoshi(2016)Clin J Am Soc Nephrol | 0.00048518 | (25.3/D/PD/D) | Pathogenic, Likely pathogenic | Likely pathogenic (PM1, PM2, PP2, PP3, PP4) |  |
| 342 | 35 | *COL4A4* | c.3577+1G>A |  | Imafuku(2017) Nephrology | 0 | NA | Likely pathogenic | Pathogenic (PVS1, PM2) |  |
| 343 | 36 | *COL4A4* | c.3687dupA | p.G1230Rfs*23 | Horinouchi(2020)Kidney360 | 0 | NA | Pathogenic | Pathogenic (PVS1, PM2) |  |
| 360 | 37 | *COL4A4* | c.81_86del | p.I29_L30del | Storey(2013)J Am Soc Nephrol | 0 | NA | Conflicting | Likely pathogenic (PM1, PM2, PM4, PP4) |  |
| 361 | 38 | *COL4A4* | c.1405G>A | p.G469R | Xiong(2015)Science | 0 | (28.2/D/PD/D) | Pathogenic, Likely pathogenic | Likely pathogenic (PM1, PM2, PP2, PP3) |  |
| 387 | 39 | *COL4A4* | c.81_86del | p.I29_L30del | Storey(2013)J Am Soc Nephrol | 0 | NA | Conflicting | Likely pathogenic (PM1, PM2, PM4, PP4) |  |
| 391 | 40 | *COL4A4* | c.1315G>A | p.G451S |  | 0 | (26.2/D/PD/D) | NA | Likely pathogenic (PM1, PM2, PP2, PP3, PP4） |  |
| 394 | 41 | *COL4A4* | c.2869G>A | p.G957R | Xiong(2015)Science | NA | (24.1/D/PD/D) | VUS | Likely pathogenic (PM1, PM2, PP1, PP3, PP4) |  |
| 407 | 42 | *COL4A3* | c.935G>T | p.G312V |  | NA | (33/D/PD/D) | NA | Likely pathogenic (PM1, PM2, PP2, PP3, PP4) |  |
| 408 | 43 | *COL4A4* | c.2242G>A | p.G748S | Papazachariou(2017) Clin Genet | 2.2286×e-5 | (22.1/D/PD/D) | Conflicting | Likely pathogenic (PM1, PM2, PP2, PP3, PP4) |  |
| 418 | 44 | *COL4A4* | c.2608G>C | p.G870R | Horinouchi(2020)Kidney360 | 2.519×e-5 | (24.5/D/PD/D) | NA | Likely pathogenic (PM1, PM2, PP3, PP4) |  |
| 420 | 45 | *COL4A3* | c.3547G>C | p.G1183R |  | NA | (23.6/D/PD/D) | NA | Likely pathogenic (PM1, PM2, PP1, PP3) |  |
| 423 | 46 | *COL4A3* | c.2602G>A | p.G868R |  | NA | (24.7/D/PD/D) | NA | Likely pathogenic (PM1, PM2, PP3, PP4) |  |
| 425 | 47 | *COL4A4* | c.594+1G>A |  | Bleyer(2022)Am J Nephrol | NA | NA | NA | Pathogenic (PVS1, PM2) |  |
| 752 | 48 | *COL4A3* | c.687+1G>A |  |  | 0 | NA | NA | Pathogenic (PVS1, PM2, PP4) |  |
| 762 | 49 | *COL4A3* | c.3464G>A | p.G1155D | Oka(2014)Pediatr Nephrol | 8.3195×e-5 | (27.2/D/PD/D) | Pathogenic | Likely pathogenic (PM1, PM2, PP2, PP3) |  |
| 776 | 50 | *COL4A3* | c.2603G>A | p.G868E | Zhou(2023)J Nephrol | 0 | (24.3/D/PD/D) | Pathogenic | Likely pathogenic (PM1, PM2, PP2, PP3, PP4) |  |
| 788 | 51 | *COL4A3* | c.1150G>C | p.G384R | Rao(2019)Clin Genet | NA | （36/D/PD/D） | NA | Likely pathogenic (PM1, PM2, PP2, PP3, PP4） |  |
| 793 | 52 | *COL4A3* | c.1900G>A | p.G634R |  | NA | (25.0/D/PD/D) | NA | Likely pathogenic (PM1, PM2, PM5, PP2, PP4, PP3) |  |
| 707 | 53 | *COL4A4* | c.1231G>A | p.G411R | Weber(2016)Pediatr Nephrol | 0 | (26.2/D/PD/D) | NA | Likely pathogenic (PM1, PM2, PP2, PP3） |  |
| 723 | 54 | *COL4A4* | c.1616G>A | p.G539E |  | NA | (25.4/D/PD/D) | NA | Likely pathogenic (PM1, PM2, PP2, PP3, PP4) |  |
| 730 | 55 | *COL4A4* | c.5057G>C | p.C1686S |  | NA | (24.5/D/PD/D) | NA | Likely pathogenic (PM1, PM2, PP2, PP3, PP4) |  |
| 734 | 56 | *COL4A4* | c.3461G>T | p.G1154V |  | NA | (25.4/D/PD/D) | NA | Likely pathogenic (PM1, PM2, PP2, PP3, PP4) |  |
| 742 | 57 | *COL4A4* | c.2269G>C | p.G757R |  | NA | (26.9/D/PD/D) | NA | Likely pathogenic (PM1, PM2, PP1, PP2, PP3, PP4) |  |
| 745 | 58 | *COL4A4* | c.3973+1G>T |  | Bleyer(2022)Am J Nephrol | NA | NA | Likely pathogenic | Pathogenic (PVS1, PM2, PP4) |  |
| 746 | 59 | *COL4A4* | c.3021C>G | p.Y1007* |  | NA | NA | NA | Pathogenic (PVS1, PM2, PP4) |  |
| 756 | 60 | *COL4A4* | c.2084G>A | p.G695D | Horinouchi(2020)Kidney360 | 2.7739×e-5 | (23.3/D/PD/D) | Likely pathogenic | Likely pathogenic (PM1, PM2, PP2, PP3) |  |
| 772 | 61 | *COL4A4* | c.2084G>A | p.G695D | Horinouchi(2020)Kidney360 | 2.7739×e-5 | (23.3/D/PD/D) | Likely pathogenic | Likely pathogenic (PM1, PM2, PP2, PP3, PP4) |  |
| 777 | 62 | *COL4A4* | c.4847T>G | p.L1616R | Imafuku(2017) Nephrology | 7.2692×e-5 | (28.9/D/PD/D) | NA | Likely pathogenic (PM1, PM2, PP2, PP3, PP4) |  |
| 778 | 63 | *COL4A4* | c.685G>C | p.G229R |  | 0 | (26.1/D/PD/D) | NA | Likely pathogenic (PM1, PM2, PP2, PP3, PP4) |  |
| 784 | 64 | *COL4A4* | c.1597G>A | p.G533S |  | 0 | (25.9/D/PD/D) | Conflicting | Likely pathogenic (PM1, PM2, PP2, PP3, PP4) |  |
| 791 | 65 | *COL4A4* | c.2608G>C | p.G870R | Horinouchi(2020)Kidney360 | 2.519×e-5 | (24.5/D/PD/D) | NA | Likely pathogenic (PM1, PM2, PP2, PP3, PP4) |  |
| 798 | 66 | *COL4A4* | c.1535G>T | p.G512V |  | NA | (26.3/D/PD/D) | NA | Likely pathogenic (PM1, PM2, PP2, PP3, PP4) |  |
| 799 | 67 | *COL4A4* | c.1535G>T | p.G512V |  | NA | (26.3/D/PD/D) | NA | Likely pathogenic (PM1, PM2, PP2, PP3, PP4) |  |
| 808 | 68 | *COL4A3* | c.1531G>A | p.G511R |  | NA | (26.1/D/PD/D) | NA | Likely pathogenic (PM1, PM2, PP2, PP3, PP4) |  |
| 836 | 69 | *COL4A3* | c.2863G>A | p.G955R | Imafuku(2017) Nephrology | 0.00011091 | (31/D/PD/D) | Likely pathogenic | Likely pathogenic (PM1, PM2, PP2, PP3) |  |
| 804 | 70 | *COL4A4* | c.4487dupT | p.E1497Gfs*22 |  | NA | NA | NA | Pathogenic (PVS1, PM2, PP4) |  |
| 809 | 71 | *COL4A4* | c.977G>A | p.G326E |  | NA | (26.2/D/PD/D) | Likely pathogenic | Likely pathogenic (PM1, PM2, PP2, PP3, PP4) |  |
| 819 | 72 | *COL4A4* | c.1579G>T | p.G527C | Marcocci(2009)Nephrol Dial Transplant | 0 | (25.9/D/PD/D) | Conflicting | Likely pathogenic (PM1, PM2, PP1, PP2, PP3) |  |
| 826 | 73 | *COL4A4* | c.2384G>A | p.G795E |  | NA | (32/D/PD/D) | NA | Likely pathogenic (PM1, PM2, PM5, PP1, PP2, PP3, PP4) |  |
| 833 | 74 | *COL4A4* | c.71+1G>A |  | Shapiro(2018)Nephrol Dial transplant | 0 | NA | Pathogenic | Pathogenic (PVS1, PM2) |  |
| 838 | 75 | *COL4A4* | c.2510G>C | p.G837A | Kamiyoshi(2016)Clin J Am Soc Nephrol | 0.00048518 | (25.3/D/PD/D) | Pathogenic, Likely pathogenic | Likely pathogenic (PM1, PM2, PP2, PP3, PP4) |  |
| 695 | 76 | *COL4A4* | c.2084G>A | p.G695R | Horinouchi(2020)Kidney360 | 2.7739×e-5 | (23.3/D/PD/D) | Likely pathogenic | Likely pathogenic (PM1, PM2, PP1, PP2, PP3, PP4) |  |
| 607 | 77 | *COL4A3* | c.1936G>A | p.G646R |  | NA | (27.7/D/PD/D) | NA | Likely pathogenic (PM1, PM2, PP2, PP3, PP4) |  |
| 610 | 78 | *COL4A3* | c.1855G>A | p.G619R | Oka(2014)Pediatr Nephrol | 2.5193×e-5 | (24.6/D/PD/D) | Pathogenic, Likely pathogenic | Likely pathogenic (PM1, PM2, PP2, PP3, PP4) |  |
| 613 | 79 | *COL4A3* | c.3134G>A | p.G1045D | Pescucci(2004)Kidney Int | NA | (28.8/D/PD/D) | NA | Likely pathogenic (PM1, PM2, PP1, PP2, PP3, PP4) |  |
| 627 | 80 | *COL4A3* | c.898G>A | p.G300R | Mencarelli(2015) J Med Genet | 0 | (28.2/D/PD/D) | Conflicting | Likely pathogenic (PM1, PM2, PP2, PP3, PP4) |  |
| 630 | 81 | *COL4A3* | c.2330G>A | p.G777D | Oka(2014)Pediatr Nephrol | NA | (29.4/D/PD/D) | NA | Likely pathogenic (PM1, PM2, PM5, PP2, PP3) |  |
| 641 | 82 | *COL4A3* | c.1909G>A | p.G637R | Zhou(2023)J Nephrol | 2.228×e-5 | (24.9/D/PD/D) | Conflicting | Likely pathogenic (PM1, PM2, PP2, PP3, PP4) |  |
| 661 | 83 | *COL4A3* | c.3874G>T | p.G1292C |  | 2.773×e-5 | (25.5/D/PD/D) | NA | Likely pathogenic (PM1, PM2, PP2, PP3) |  |
| 668 | 84 | *COL4A3* | c.469G>C | p.G157R | Zhang(2021)Pediatr Nephrol | 4.4621×e-5 | (33/D/PD/D) | VUS | Likely pathogenic (PM1, PM2, PP2, PP4) |  |
| 693 | 85 | *COL4A3* | c.2980G>C | p.G994R |  | NA | (35/D/PD/D) | NA | Likely pathogenic (PM1, PM2, PP2, PP3) |  |
| 619 | 86 | *COL4A4* | c.870G>A | p.Lys290= | Sun(2018)Sci Rep | 0 | NA | Conflicting | Pathogenic (PVS1, PM2 ) |  |
| 631 | 87 | *COL4A4* | c.755G>T | p.G252V | Bullich(2018)Kidney Int | 0 | (25.8/D/PD/D) | Likely pathogenic | Likely pathogenic (PM1, PM2, PP2, PP3, PP4) |  |
| 642 | 88 | *COL4A4* | c.2084G>A | p.G695D | Horinouchi(2020)Kidney360 | 2.7739×e-5 | (23.3/D/PD/D) | Likely pathogenic | Likely pathogenic (PM1, PM2, PP2, PP3, PP4) |  |
| 645 | 89 | *COL4A4* | c.4216+1G>A |  |  | 0 | NA | NA | Pathogenic (PVS1, PM2, PP4) |  |
| 648 | 90 | *COL4A4* | c.1323_1340del | p.P444_L449del | Boye(1998)Am J Hum Genet | 8.9099×e^-5^ | NA | Pathogenic, Likely pathogenic | Likely pathogenic (PM2, PM4, PP1, PP4） |  |
| 655 | 91 | *COL4A4* | c.2084G>A | p.G695D | Horinouchi(2020)Kidney360 | 2.7739×e-5 | (23.3/D/PD/D) | Likely pathogenic | Likely pathogenic (PM1, PM2, PP2, PP3, PP4) |  |
| 675 | 92 | *COL4A4* | c.71+1G>A |  | Shapiro(2018)Nephrol Dial transplant | 0 | NA | Pathogenic | Pathogenic (PVS1, PM2, PP4) |  |
| 683 | 93 | *COL4A4* | c.2641del | p.H881Mfs*69 | Kamiyoshi(2016)Clin J Am Soc Nephrol | NA | NA | NA | Pathogenic (PVS1, PM2) |  |
| 504 | 94 | *COL4A3* | c.2863G>A | p.G955R | Imafuku(2017) Nephrology | 0.00011091 | (31/D/PD/D) | Likely pathogenic | Likely pathogenic (PM1, PM2, PP2, PP3, PP4) |  |
| 522 | 95 | *COL4A3* | c.1354G>A | p.G452R | Oka(2014)Pediatr Nephrol | 0 | (24.8/D/PD/D) | Conflicting | Likely pathogenic (PM1, PM2, PP2, PP3） |  |
| 544 | 96 | *COL4A3* | c.388-1G>C |  |  | NA | NA | NA | Pathogenic (PVS1, PM2) |  |
| 563 | 97 | *COL4A3* | c.469G>C | p.G157R | Zhang(2021)Pediatr Nephrol | 4.4621×e-5 | (33/D/PD/D) | VUS | Likely pathogenic (PM1, PM2, PP2, PP4) |  |
| 512 | 98 | *COL4A4* | c.71+1G>A |  | Shapiro(2018)Nephrol Dial transplant | 0 | NA | Pathogenic | Pathogenic (PVS1, PM2, PP4) |  |
| 518 | 99 | *COL4A4* | c.2510G>C | p.G837A | Kamiyoshi(2016)Clin J Am Soc Nephrol | 0.00048518 | (25.3/D/PD/D) | Pathogenic, Likely pathogenic | Likely pathogenic (PM1, PM2, PP3, PP4) |  |
| 523 | 100 | *COL4A4* | c.3307G>A | p.G1103R | Horinouchi(2020)Kidney360 | 0.00015596 | (24.6/D/PD/D) | Conflicting | Likely pathogenic (PS1, PM1, PM2, PP2, PP3) |  |
| 531 | 101 | *COL4A4* | c.2084G>A | p.G695D | Horinouchi(2020)Kidney360 | 2.7739×e-5 | (23.3/D/PD/D) | Likely pathogenic | Likely pathogenic (PS1, PM1, PM2, PP2, PP3, PP4) |  |
| 538 | 102 | *COL4A4* | c.1862dupC | p.G622Rfs*35 |  | 0 | NA | NA | Pathogenic (PVS1, PM2, PP4) |  |
| 542 | 103 | *COL4A4* | c.1498_1499del | p.G500Pfs*14 |  | NA | NA | NA | Pathogenic (PVS1, PM2, PP4） |  |
| 543 | 104 | *COL4A4* | c.1498_1499del | p.G500Pfs*14 |  | NA | NA | NA | Pathogenic (PVS1, PM2, PP4） |  |
| 547 | 105 | *COL4A4* | c.2084G>A | p.G695D | Horinouchi(2020)Kidney360 | 2.7739×e-5 | (23.3/D/PD/D) | Likely pathogenic | Likely pathogenic (PM1, PM2, PP2, PP3, PP4) |  |
| 551 | 106 | *COL4A4* | c.2510G>C | p.G837A | Kamiyoshi(2016)Clin J Am Soc Nephrol | 0.00048518 | (25.3/D/PD/D) | Pathogenic, Likely pathogenic | Likely pathogenic (PM1, PM2, PP2, PP3, PP4) |  |
| 565 | 107 | *COL4A4* | c.2084G>A | p.G695D | Horinouchi(2020)Kidney360 | 2.7739×e-5 | (23.3/D/PD/D) | Likely pathogenic | Likely pathogenic (PM1, PM2, PP2, PP3, PP4) |  |
| 568 | 108 | *COL4A4* | c.2617G>A | p.G873R | Horinouchi(2020)Kidney360 | 0 | (24.2/D/PD/D) | NA | Likely pathogenic (PM1, PM2, PP1, PP2, PP3, PP4) |  |
| 580 | 109 | *COL4A4* | c.3179G>A | p.G1060E |  | NA | (26.7/D/PD/D) | NA | Likely pathogenic (PM1, PM2, PP2, PP3) |  |
| 586 | 110 | *COL4A4* | c.735G>A | p.P245= | Sun(2018)Int J Clin Exp Pathol | 4.4553×e-5 | NA | Pathogenic, Likely pathogenic | Pathogenic (PVS1, PM2, PP4,) |  |
| 590 | 111 | *COL4A4* | c.327+1G>A |  |  | 0 | NA | NA | Pathogenic (PVS1, PM2, PP4) |  |
| 591 | 112 | *COL4A4* | c.4809+5G>A |  |  | 0 | NA | NA | Pathogenic (PVS1, PM2, PP1) |  |
| 600 | 113 | *COL4A4* | c.1579G>T | p.G527C | Marcocci(2009)Nephrol Dial Transplant | 0 | (25.9/D/PD/D) | Conflicting | Likely pathogenic (PM1, PM2, PP2, PP3, PP4) |  |
| 433 | 114 | *COL4A3* | c.1918G>A | p.G640R | Horinouchi(2020)Kidney360 | 0 | (23.7/D/PD/D) | Pathogenic, Likely pathogenic | Likely pathogenic (PM1, PM2, PP2, PP3, PP4) |  |
| 437 | 115 | *COL4A3* | c.2863G>A | p.G955R | Imafuku(2017) Nephrology | 0.00011091 | (31/D/PD/D) | Likely pathogenic | Likely pathogenic (PM1, PM2, PP2, PP3, PP4) |  |
| 451 | 116 | *COL4A3* | c.1826delC | p.P609Lfs*138 |  | NA | NA | NA | Pathogenic (PVS1, PM2, PP4) |  |
| 457 | 117 | *COL4A3* | c.833dupT | p.P279Afs*8 |  | 2.5208×e-5 | NA | Pathogenic, Likely pathogenic | Pathogenic (PVS1, PM2) |  |
| 461 | 118 | *COL4A3* | c.1909G>A | p.G637R | Zhou(2023)J Nephrol | 2.228×e-5 | (24.9/D/PD/D) | Conflicting | Likely pathogenic (PM1, PM2, PP2, PP3) |  |
| 466 | 119 | *COL4A3* | c.997G>A | p.G333R |  | 0 | (26.4/D/PD/D) | Conflicting | Likely pathogenic (PM1, PM2, PP2, PP4, PP3) |  |
| 477 | 120 | *COL4A3* | c.991C>T | p.Q331* |  | 0 | NA | Likely pathogenic | Pathogenic (PVS1, PM2, PP4) |  |
| 434 | 121 | *COL4A4* | c.4421C>G | p.T1474R |  | NA | (26.4,/D/PD/D) | NA | Likely pathogenic (PM1, PM2, PP2, PP3, PP4) |  |
| 439 | 122 | *COL4A4* | c.695G>A | p.G232E |  | 0 | (30/D/PD/D) | NA | Likely pathogenic (PM1, PM2, PP2, PP3, PP4) |  |
| 441 | 123 | *COL4A4* | c.4333+2T>C |  | Sun(2018)Sci Rep | 0 | NA | Likely pathogenic | Pathogenic (PVS1, PM2) |  |
| 444 | 124 | *COL4A4* | c.4768C>T | p.Q1590* | Groopman(2019)N Eng J Med | NA | NA | Pathogenic | Pathogenic (PVS1, PS1, PM2) |  |
| 445 | 125 | *COL4A4* | c.2743G>C | p.G915R | Yuan(2022)J Am Soc Nephrol | 0 | (24.8/D/PD/D) | NA | Likely pathogenic (PM1, PM2, PP2, PP3) |  |
| 453 | 126 | *COL4A4* | c.1666A>G | p.K556E |  | 0.00085651 | (24.4/B/PD/B) | Uncertain significance | Likely pathogenic (PM1, PM2, PP2, PP4) |  |
| 464 | 127 | *COL4A4* | c.4421C>T | p.T1474M | Zhao(2019)Mol Genet Genomic | 0.0010473 | (26.5/ D/PD/D) | Conflicting | Likely pathogenic (PM1, PM2, PP2, PP3, PP4,) |  |
| 474 | 128 | *COL4A4* | c.1733G>T | p.G578V | Horinouchi(2020)Kidney360 | NA | (25.7/D/PD/D) | Pathogenic | Likely pathogenic (PM1, PM2, PP2, PP3, PP4) |  |
| 495 | 129 | *COL4A4* | c.71+1G>A |  | Shapiro(2018)Nephrol Dial transplant | 0 | NA | Pathogenic | Pathogenic (PVS1, PM2, PP4) |  |
| 1104 | 130 | *COL4A3* | c.469G>C | p.G157R | Zhang(2021)Pediatr Nephrol | 4.4621×e-5 | (33/D/PD/D) | VUS | Likely pathogenic (PM1, PM2, PP2, PP4) |  |
| 1108 | 131 | *COL4A3* | c.2981-2 A>C |  |  | NA | NA | NA | Pathogenic (PVS1, PM2) |  |
| 1111 | 132 | *COL4A3* | c.3464G>A | p.G1155D | Oka(2014)Pediatr Nephrol | 8.3195×e-5 | (27.2/D/PD/D) | Pathogenic | Likely pathogenic (PM1, PM2, PP1, PP2, PP3, PP4) |  |
| 1115 | 133 | *COL4A3* | c.2620G>C | p.G874R |  | NA | (26.4/D/PD/D) | NA | Likely pathogenic (PM1, PM2, PP1, PP2, PP3, PP4) |  |
| 1135 | 134 | *COL4A3* | c.469G>C | p.G157R | Zhang(2021)Pediatr Nephrol | 4.4621×e-5 | (33/D/PD/D) | VUS | Likely pathogenic (PM1, PM2, PP2, PP4) |  |
| 1137 | 135 | *COL4A3* | c.3427G>A | p.G1143R | Horinouchi(2020)Kidney360 | NA | (28.0/D/PD/D) | NA | Likely pathogenic (PM1, PM2, PP1, PP2, PP3, PP4) |  |
| 1139 | 136 | *COL4A3* | c.4028-1G>A |  |  | 0 | NA | NA | Pathogenic (PVS1, PM2) |  |
| 1143 | 137 | *COL4A3* | c.1576-20_1576-6del |  |  | NA | NA | NA | Pathogenic (PVS1, PM2) |  |
| 1157 | 138 | *COL4A3* | c.2330G>A | p.G777D | Oka(2014)Pediatr Nephrol | NA | (29.4/D/PD/D) | NA | Likely pathogenic (PM1, PM2, PM5, PP1, PP2, PP3, PP4) |  |
| 1173 | 139 | *COL4A3* | c.1087G>A | p.G363R |  | 0 | (27.4/D/PD/D) | Likely pathogenic | Likely Pathogenic (PM1, PM2, PP2, PP3） |  |
| 1177 | 140 | *COL4A3* | c.1855G>A | p.G619R | Oka(2014)Pediatr Nephrol | 2.5193×e-5 | (24.6/D/PD/D) | Pathogenic, Likely pathogenic | Likely pathogenic (PM1, PM2, PP2, PP3) |  |
| 1187 | 141 | *COL4A3* | c.3266G>A | p.G1089D | Oka(2014)Pediatr Nephrol | NA | (25.9/D/PD/D) | Pathogenic | Likely pathogenic (PM1, PM2, PP2, PP3) |  |
| 1188 | 142 | *COL4A3* | c.1909G>A | p.G637R | Zhou(2023)J Nephrol | 2.228×e-5 | (24.9/D/PD/D) | Conflicting | Likely pathogenic (PM1, PM2, PP2, PP3) |  |
| 1191 | 143 | *COL4A3* | c.2863G>A | p.G955R | Imafuku(2017) Nephrology | 0.00011091 | (31/D/PD/D) | Likely pathogenic | Likely pathogenic (PM1, PM2, PP2, PP3) |  |
| 1198 | 144 | *COL4A3* | c.1751G>A | p.G584D |  | NA | (26.7/D/PD/D) | NA | Likely Pathogenic (PM1, PM2, PM5, PP2, PP3) |  |
| 1201 | 145 | *COL4A3* | c.953G>A | p.G318D | Horinouchi(2020)Kidney360 | 0 | (25.7/D/PD/D) | Likely pathogenic | Likely pathogenic (PM1, PM2, PP2, PP3) |  |
| 1203 | 146 | *COL4A3* | c.898G>A | p.G300R | Mencarelli(2015) J Med Genet | 0 | (28.2/D/PD/D) | Conflicting | Likely pathogenic (PM1, PM2, PP2, PP3) |  |
| 1211 | 147 | *COL4A3* | c.2981-2A>C |  |  | NA | NA | NA | Pathogenic (PVS1, PM2) |  |
| 1212 | 148 | *COL4A3* | c.908G>T | p.G303V |  | NA | (25.2/D/PD/D) | NA | Likely pathogenic (PM1, PM2, PP1, PP2, PP3) |  |
| 1030 | 149 | *COL4A3* | c.3079G>C | p.G1027R |  | NA | (28.4/D/PD/D) | NA | Likely pathogenic (PM1, PM2, PP1, PP2, PP3, PP4) |  |
| 1041 | 150 | *COL4A3* | c.2567G>A | p.G856E | Storey(2013)J Am Soc Nephrol | NA | (26.0/D/PD/D) | NA | Likely pathogenic (PM1, PM2, PP2, PP3) |  |
| 1044 | 151 | *COL4A3* | c.2125G>T | p.G709* | Oka(2014)Pediatr Nephrol | NA | NA | NA | Pathogenic (PVS1, PM2) |  |
| 1046 | 152 | *COL4A3* | c.1855G>A | p.G619R | Oka(2014)Pediatr Nephrol | 2.5193×e-5 | (24.6/D/PD/D) | Pathogenic, Likely pathogenic | Likely pathogenic (PM1, PM2, PP2, PP3) |  |
| 1049 | 153 | *COL4A3* | c.2863G>A | p.G955R | Imafuku(2017) Nephrology | 0.00011091 | (31/D/PD/D) | Likely pathogenic | Likely pathogenic (PM1, PM2, PP2, PP3) |  |
| 1069 | 154 | *COL4A3* | c.2567G>A | p.G856E | Storey(2013)J Am Soc Nephrol | NA | (26.0/D/PD/D) | NA | Likely pathogenic (PM1, PM2, PP2, PP3) |  |
| 1072 | 155 | *COL4A3* | c.3592G>C | p.G1198R | Groopman(2019)N Eng J Med | NA | (24.5/D/PD/D) | NA | Likely pathogenic (PM1, PM2 PP2, PP3) |  |
| 1078 | 156 | *COL4A3* | c.2320G>A | p.G777D |  | NA | (29.4/D/PD/D) | NA | Likely pathogenic (PM1, PM2, PP2, PP3, PP4) |  |
| 1081 | 157 | *COL4A3* | c.4793T>G | p.L1598R | Oka(2014)Pediatr Nephrol | 0.00075754 | (28.4/D/PD/D) | Conflicting | Likely pathogenic (PM1, PM2, PP2, PP3) |  |
| 1096 | 158 | *COL4A3* | c.3427G>A | p.G1143R | Horinouchi(2020)Kidney360 | NA | (28.0/D/PD/D) | NA | Pathogenic (PM1, PM2, PP1, PP2, PP3) |  |
| 902 | 159 | *COL4A3* | c.469G>C | p.G157R | Zhang(2021)Pediatr Nephrol | 4.4621×e-5 | (33/D/PD/D) | VUS | Likely pathogenic (PM1, PM2, PP2, PP4) |  |
| 920 | 160 | *COL4A3* | c.1504+1G>A | - |  | 0 | NA | Pathogenic, Likely pathogenic | Pathogenic (PVS1, PM2, PM5） |  |
| 922 | 161 | *COL4A3* | c.548G>A | p.G183R | Domingo-Gallego(2022) Nephrol Dial Transplant | 0 | (32/D/PD/D) | NA | Likely pathogenic (PM1, PM2, PP1, PP2, PP3 PP4) |  |
| 923 | 162 | *COL4A3* | c.469G>C | p.G157R | Zhang(2021)Pediatr Nephrol | 4.4621×e-5 | (33/D/PD/D) | VUS | Likely pathogenic (PM1, PM2, PP2, PP4) |  |
| 931 | 163 | *COL4A3* | c.2330G>A | p.G777D | Oka(2014)Pediatr Nephrol | NA | (29.4/D/PD/D) | NA | Likely pathogenic (PM1, PM2, PP2, PP3) |  |
| 934 | 164 | *COL4A3* | c.3427G>A | p.G1143R | Horinouchi(2020)Kidney360 | NA | (28.0/D/PD/D) | NA | Likely pathogenic (PM1, PM2, PP2, PP3) |  |
| 940 | 165 | *COL4A3* | c.2125G>C | p.G709R |  | NA | (34/D/PD/D) | NA | Likely pathogenic (PM1, PM2, PP3, PP4) |  |
| 944 | 166 | *COL4A3* | c.3464G>A | p.G1155D | Oka(2014)Pediatr Nephrol | 8.3195×e-5 | (27.2/D/PD/D) | Pathogenic | Likely pathogenic (PM1, PM2, PP2, PP3) |  |
| 949 | 167 | *COL4A3* | c.646-1G>A |  |  | NA | NA | NA | Pathogenic (PVS1, PM2) |  |
| 956 | 168 | *COL4A3* | c.3464G>A | p.G1155D | Oka(2014)Pediatr Nephrol | 8.3195×e-5 | (27.2/D/PD/D) | Pathogenic | Likely pathogenic (PM1, PM2, PP2, PP3) |  |
| 959 | 169 | *COL4A3* | c.2728G>A | p.G910S |  | 2.5189×e-5 | (24.6/D/PD/A) | NA | Likely pathogenic (PM1, PM2, PP2. PP3) |  |
| 966 | 170 | *COL4A3* | c.1918G>A | p.G640R | Horinouchi(2020)Kidney360 | 0 | (23.7/D/PD/D) | Pathogenic, Likely pathogenic | Likely pathogenic (PM1, PM2, PP1, PP2, PP3, PP4) |  |
| 971 | 171 | *COL4A3* | c.1604G>T | p.G535V | Gao(2022)Front Genet | NA | (27.0/D/PD/D) | NA | Likely pathogenic (PM1, PM2, PP2, PP3) |  |
| 985 | 172 | *COL4A3* | c.2065G>C | p.G689R | Moriniera(2014)J Am Soc Nephrol | NA | (26.9/D/PD/D) | NA | Likely Pathogenic (PM1, PM2, PP1, PP2, PP3) |  |
| 986 | 173 | *COL4A3* | c.3160G>A | p.G1054R |  | 0 | (29.6/D/PD/D) | NA | Likely pathogenic (PM1, PM2, PP1, PP2, PP3) |  |
| 849 | 174 | *COL4A3* | c.1354G>A | p.G452R | Oka(2014)Pediatr Nephrol | 0 | (24.8/D/PD/D) | Conflicting | Likely pathogenic (PM1, PM2, PP2, PP3） |  |
| 867 | 175 | *COL4A3* | c.2330G>A | p.G777D | Oka(2014)Pediatr Nephrol | NA | (29.4/D/PD/D) | NA | Likely pathogenic (PM1, PM2, PP2, PP3) |  |
| 875 | 176 | *COL4A3* | c.655G>T | p.G219C |  | 7.5582×e-5 | (28.1/D/PD/D) | NA | Likely pathogenic (PM1, PM2, PP2, PP3) |  |
| 877 | 177 | *COL4A3* | c.2029G>A | p.G677R |  | NA | (28.3/D/PD/D) | NA | Likely pathogenic (PM1, PM2, PP2, PP3) |  |
| 879 | 178 | *COL4A3* | c.916G>A | p.G306S |  | NA | (32/D/PD/D) | NA | Likely pathogenic (PM1, PM2, PP2, PP3) |  |
| 883 | 179 | *COL4A3* | c.2819G>T | p.G940V |  | NA | (25.3/D/PD/D) | NA | Likely pathogenic (PM1, PM2, PP2, PP3) |  |
| 885 | 180 | *COL4A3* | c.3134G>A | p.G1045D | Pescucci(2004)Kidney Int | NA | (28.8/D/PD/D) | NA | Likely pathogenic (PM1, PM2, PP1, PP2, PP3) |  |
| 889 | 181 | *COL4A3* | c.1883G>A | p.G628D | Al-Hmed(2022)Hum Mutat | 2.5189×e-5 | （24.1/D/PD/D) | VUS | Likely pathogenic (PM1, PM2, PP2, PP3) |  |
| 1110 | 182 | *COL4A4* | c.2566C>T | p.Q856* | Horinouchi(2020)Kidney360 | NA | NA | NA | Pathogenic (PVS1, PM2) |  |
| 1120 | 183 | *COL4A4* | c.5044C>T | p.R1682W | Yuan(2022)J Am Soc Nephrol | 0 | (23.9/D/PD/D) | VUS | Likely pathogenic (PM1, PM2, PP2, PP3) |  |
| 1121 | 184 | *COL4A4* | c.2641delC | p.H881Mfs*69 | Kamiyoshi(2016)Clin J Am Soc Nephrol | NA | NA | NA | Pathogenic (PVS1, PM2, PP1, PP4) |  |
| 1123 | 185 | *COL4A4* | c.4079delC | p.P1360Rfs*28 |  | 0 | NA | Pathogenic | Pathogenic (PVS1, PM2) |  |
| 1128 | 186 | *COL4A4* | c.2752G>A | p.G918R | Fallerini(2014)Clin Genet | 0.00011136 | (24.5/D/PD/D) | Conflicting | Likely pathogenic (PM1, PM2, PP1, PP2, PP3, PP4) |  |
| 1129 | 187 | *COL4A4* | c.4495C>T | p.G1499* |  | NA | NA | NA | Pathogenic (PVS1, PM2) |  |
| 1130 | 188 | *COL4A4* | c.4421C>T | p.T1474M | Zhao(2019)Mol Genet Genomic | 0.0010473 | (26.5/ D/PD/D) | Conflicting | Likely pathogenic (PM2, PP1, PP2, PP3, PP4) |  |
| 1131 | 189 | *COL4A4* | c.4847T>G | p.L1616R | Imafuku(2017) Nephrology | 7.2692×e-5 | (28.9/D/PD/D) | NA | Likely pathogenic (PM1, PM2, PP2, PP3, PP4) |  |
| 1151 | 190 | *COL4A4* | c.4332A>G | p.P1444= |  | NA | NA | NA | Pathogenic (PVS1, PM2) |  |
| 1158 | 191 | *COL4A4* | c.596_619del | p.G199_P206del |  | NA | NA | NA | Likely pathogenic (PM1, PM2, PM4) |  |
| 1159 | 192 | *COL4A4* | c.4333+1G>A |  |  | NA | NA | NA | Pathogenic (PVS1, PM2) |  |
| 1160 | 193 | *COL4A4* | c.1579G>T | p.G527C |  | 0 | (25.9/D/PD/D) | Conflicting | Likely pathogenic (PM1, PM2, PP2, PP3) |  |
| 1161 | 194 | *COL4A4* | c.2510G>C | p.G837A | Kamiyoshi(2016)Clin J Am Soc Nephrol | 0.00048518 | (25.3/D/PD/D) | Pathogenic, Likely pathogenic | Likely pathogenic (PM1, PM2, PP2, PP3) |  |
| 1171 | 195 | *COL4A4* | c.1108G>C | p.G370R | Moriniera(2014)J Am Soc Nephrol | 2.77×e^-5^ | (23.0/D/PD/D) | Likely pathogenic | Likely pathogenic (PM1, PM2, PP1, PP2, PP3） |  |
| 1172 | 196 | *COL4A4* | c.1144G>C | p.G382R | Rao(2019)Clin Genet | NA | (23.5/D/PD/D) | NA | Likely pathogenic (PM1, PM2, PP1, PP2, PP3） |  |
| 1185 | 197 | *COL4A4* | c.4564_4568delTTTGC | p.F1522Lfs*33 |  | NA | NA | NA | Pathogenic (PVS1, PM2) |  |
| 1189 | 198 | *COL4A4* | c.4847T>G | p.L1616R | Imafuku(2017) Nephrology | 7.2692×e-5 | (28.9/D/PD/D) | NA | Likely pathogenic (PM1, PM2, PP2, PP3, PP4) |  |
| 1192 | 199 | *COL4A4* | c.4421C>T | p.T1474M | Zhao(2019)Mol Genet Genomic | 0.0010473 | (26.5/ D/PD/D) | Conflicting | Likely pathogenic (PM1, PM2, PP2, PP3, PP4, ) |  |
| 1195 | 200 | *COL4A4* | c.827G>C | p.G276A | Imafuku(2017) Nephrology | 0.00089806 | (25.5/D/PD/D) | Likely pathogenic | Likely pathogenic (PM1, PM2, PP2, PP3) |  |
| 1210 | 201 | *COL4A4* | c.4421C>T | p.T1474M | Zhao(2019)Mol Genet Genomic | 0.0010473 | (26.5/ D/PD/D) | Conflicting | Likely pathogenic (PM1, PM2, PP2, PP3, PP4, ) |  |
| 1216 | 202 | *COL4A4* | c.1396G>A | p.G466R | Weber(2016) Pediatr Nephrol | 0 | (26.6/D/PD/D) | Pathogenic, Likely pathogenic | Likely pathogenic (PM1, PM2, PP2, PP3) |  |
| 1002 | 203 | *COL4A4* | c.2084G>A | p.G695D | Horinouchi(2020)Kidney361 | 2.7739×e-5 | (23.3/D/PD/D) | Likely pathogenic | Likely pathogenic (PS1, PM1, PM2, PP2, PP3) |  |
| 1011 | 204 | *COL4A4* | c.2510G>C | p.G837A | Kamiyoshi(2016)Clin J Am Soc Nephrol | 0.00048518 | (25.3/D/PD/D) | Pathogenic, Likely pathogenic | Likely pathogenic (PM1, PM2, PP2, PP3) |  |
| 1012 | 205 | *COL4A4* | c.4073G>A | p.G1358D |  | NA | (24.3/D/PD/D) | NA | Likely pathogenic (PM1, PM2, PP2, PP3) |  |
| 1013 | 206 | *COL4A4* | c.2084G>A | p.G695D | Horinouchi(2020)Kidney362 | 2.7739×e-5 | (23.3/D/PD/D) | Likely pathogenic | Likely pathogenic (PM1, PM2, PP2, PP3) |  |
| 1016 | 207 | *COL4A4* | c.2878G>A | p.G960R | Horinouchi(2020)Kidney360 | 0 | (25.4/D/PD/D) | Pathogenic, Likely pathogenic | Likely pathogenic (PM1, PM2, PP2, PP3) |  |
| 1028 | 208 | *COL4A4* | c.4847T>G | p.L1616R | Imafuku(2017) Nephrology | 7.2692×e-5 | (28.9/D/PD/D) | NA | Likely pathogenic (PM1, PM2, PP2, PP3, PP4) |  |
| 1029 | 209 | *COL4A4* | c.2681G>A | p.G894E |  | NA | (23.7/D/PD/D) | NA | Likely pathogenic (PM1, PM2, PP2, PP3) |  |
| 1035 | 210 | *COL4A4* | c.2384G>A | p.G795E |  | NA | (32/D/PD/D) | NA | Likely pathogenic (PM1, PM2, PM5, PP1, PP2, PP3) |  |
| 1048 | 211 | *COL4A4* | c.71+1 G>A |  | Shapiro(2018)Nephrol Dial transplant | 0 | NA | Pathogenic | Pathogenic (PVS1, PS1, PM2) |  |
| 1053 | 212 | *COL4A4* | c.1396G>A | p.G466R | Weber(2016) Pediatr Nephrol | 0 | (26.6/D/PD/D) | Pathogenic, Likely pathogenic | Likely pathogenic (PM1, PM2, PP2, PP3) |  |
| 1055 | 213 | *COL4A4* | c.4129C>T | p.R1377* | Boye(1998)Am J Hum Genet | 0 | NA | Pathogenic | Pathogenic (PVS1, PM2, PP1) |  |
| 1057 | 214 | *COL4A4* | c.2510G>C | p.G837A | Kamiyoshi(2016)Clin J Am Soc Nephrol | 0.00048518 | (25.3/D/PD/D) | Pathogenic, Likely pathogenic | Likely pathogenic (PM1, PM2, PP2, PP3) |  |
| 1058 | 215 | *COL4A4* | c.3021C>G | p.Y1007* |  | NA | NA | NA | Pathogenic (PVS1, PM2) |  |
| 1067 | 216 | *COL4A4* | c.2084G>A | p.G695D | Horinouchi(2020)Kidney360 | 2.7739×e-5 | (23.3/D/PD/D) | Likely pathogenic | Likely pathogenic (PM1, PM2, PP1, PP2, PP3, PP4) |  |
| 1079 | 217 | *COL4A4* | c.2242G>A | p.G748S | Papazachariou(2017) Clin Genet | 2.2286×e-5 | (22.1/D/PD/D) | Conflicting | Likely pathogenic (PM1, PM2, PP2, PP3, PP4) |  |
| 1080 | 218 | *COL4A4* | c.737G>A | p.G246D |  | NA | (27.9/D/PD/D) | VUS | Likely pathogenic (PM1, PM2, PP2, PP3) |  |
| 1087 | 219 | *COL4A4* | c.4847T>G | p.L1616R | Imafuku(2017) Nephrology | 7.2692×e-5 | (28.9/D/PD/D) | NA | Likely pathogenic (PM1, PM2, PP2, PP3, PP4) |  |
| 1094 | 220 | *COL4A4* | c.1969G>A | p.G657S |  | NA | (26.4/D/PD/D) | NA | Likely pathogenic (PM1, PM2, PP2, PP3, PP4) |  |
| 1097 | 221 | *COL4A4* | c.1807dupG | p.D603Gfs*3 |  | NA | NA | NA | Pathogenic (PVS1, PM2, PP1, PP4） |  |
| 916 | 222 | *COL4A4* | c.3086delC | p.P1029Qfs*9 |  | NA | NA | NA | Pathogenic (PVS1, PM2, PP1, PP4) |  |
| 851 | 223 | *COL4A4* | c.3004G>T | p.G1002R |  | NA | NA | NA | Pathogenic (PVS1, PM2) |  |
| 871 | 224 | *COL4A4* | c.2084G>A | p.G695D | Horinouchi(2020)Kidney364 | 2.7739×e-5 | (23.3/D/PD/D) | Likely pathogenic | Likely pathogenic (PM1, PM2, PP2, PP3) |  |
| 872 | 225 | *COL4A4* | c.2084G>A | p.G695D | Horinouchi(2020)Kidney365 | 2.7739×e-5 | (23.3/D/PD/D) | Likely pathogenic | Likely pathogenic (PM1, PM2, PP1, PP2, PP3, PP4) |  |
| 874 | 226 | *COL4A4* | c.3724G>C | p.G1242R |  | NA | (25.2/D/PD/D) | NA | Likely pathogenic (PM1, PM2, PP2, PP3, PP4) |  |
| 918 | 227 | *COL4A4* | c.4847T>G | p.L1616R | Imafuku(2017) Nephrology | 7.2692×e-5 | (28.9/D/PD/D) | NA | Likely pathogenic (PM1, PM2, PP2, PP3, PP4) |  |
| 927 | 228 | *COL4A4* | c.1979G>T | p.G660V |  | NA | (25.6/D/PD/D) | NA | Likely pathogenic (PM1, PM2, PP2, PP3) |  |
| 929 | 229 | *COL4A4* | c.2879G>C | p.G960A |  | 0 | (24.7/D/PD/D) | NA | Likely pathogenic (PM1, PM2, PP2, PP3, PP4) |  |
| 937 | 230 | *COL4A4* | c.2510G>C | p.G837A | Kamiyoshi(2016)Clin J Am Soc Nephrol | 0.00048518 | (25.3/D/PD/D) | Pathogenic, Likely pathogenic | Likely pathogenic (PM1, PM2, PP1, PP2, PP3, PP4) |  |
| 957 | 231 | *COL4A4* | c.2510G>C | p.G837A | Kamiyoshi(2016)Clin J Am Soc Nephrol | 0.00048518 | (25.3/D/PD/D) | Pathogenic, Likely pathogenic | Likely pathogenic (PM1, PM2, PP2, PP3) |  |
| 961 | 232 | *COL4A4* | c.2510G>C | p.G837A | Kamiyoshi(2016)Clin J Am Soc Nephrol | 0.00048518 | (25.3/D/PD/D) | Pathogenic, Likely pathogenic | Likely pathogenic (PM1, PM2, PP2, PP3) |  |
| 970 | 233 | *COL4A4* | c.265G>T | p.G89* |  | NA | NA | NA | Pathogenic (PVS1, PM2) |  |
| 973 | 234 | *COL4A4* | c.1803+1G>A |  |  | NA | NA | NA | Pathogenic (PVS1, PM2) |  |
| 975 | 235 | *COL4A4* | c.3021C>G | p.Y1007* |  | NA | NA | NA | Pathogenic (PVS1, PM2) |  |
| 976 | 236 | *COL4A4* | c.2084G>A | p.G695D | Horinouchi(2020)Kidney363 | 2.7739×e-5 | (23.3/D/PD/D) | Likely pathogenic | Likely pathogenic (PM1, PM2, PP2, PP3) |  |
| 978 | 237 | *COL4A4* | c.623G>A | p.G208D |  | 0 | (23.7/D/PD/D) | NA | Likely pathogenic (PM1, PM2, PP1, PP2, PP4, PP3) |  |
| 979 | 238 | *COL4A4* | c.482G>A | p.G161D |  | 0 | (23.5/D/PD/D) | NA | Likely pathogenic (PM1, PM2, PP2, PP3, PP4) |  |
| 983 | 239 | *COL4A4* | c.827G>C | p.G276A | Imafuku(2017) Nephrology | 0.00089806 | (25.5/D/PD/D) | Likely pathogenic | Likely pathogenic (PM1, PM2, PP2, PP3) |  |
| 988 | 240 | *COL4A4* | c.2563G>C | p.G855R |  | NA | (24.9/D/PD/D) | NA | Likely pathogenic (PM1, PM2, PP2, PP3, PP4) |  |
| 994 | 241 | *COL4A4* | c.4129C>T | p.R1377* | Boye(1998)Am J Hum Genet | 0 | NA | Pathogenic | Pathogenic (PVS1, PM2) |  |
| 999 | 242 | *COL4A4* | c.896G>A | p.G299E |  | NA | (24.2/D/PD/D) | NA | Likely pathogenic (PM1, PM2, PP2, PP3, PP4) |  |
| A1218 | 243 | *COL4A3* | c.424G>A | p.G142S | Fujimaru(2024)Kidney Int Rep | 0.00010081 | (24.8/D/PD/D) | NA | Likely pathogenic (PM1, PM2, PP2, PP3) |  |
| A1219 | 244 | *COL4A3* | c.388-10T>G |  |  | NA | NA | NA | Pathogenic (PVS1, PM2） |  |
| A1232 | 245 | *COL4A4* | c.2617G>A | p.G873R | Horinouchi(2020)Kidney360 | 0 | (24.2/D/PD/D) | NA | Likely pathogenic (PM1, PM2, PP2, PP3, PP4) |  |
| A1234 | 246 | *COL4A4* | c.1181G>A | p.G394D | Poleitner(2023)Front Genet | NA | (23.5/D/PD/D) | NA | Likely pathogenic (PM1, PM2, PP2, PP3, PP4) |  |
| A1236 | 247 | *COL4A3* | c.1132G>A | p.G378R |  | NA | (33.0/B/PD/D) | Pathogenic, Likely pathogenic | Likely pathogenic~~VUS~~ (PM1, PM2, PP2, PP3, PP4, ) |  |
| A1238 | 248 | *COL4A3* | c.3472G>A | p.G1158R | Storey(2013)J Am Soc Nephrol | NA | （29.4/D/PD/D) | NA | Likely pathogenic (PM1, PM2, PP2, PP3, PP4) |  |
| A1239 | 249 | *COL4A4* | c.975+4A>G |  |  | NA | NA | NA | Pathogenic (PVS1, PM2) |  |
| A1240 | 250 | *COL4A3* | c.2863G>A | p.G955R | Imafuku(2017) Nephrology | 0.00011091 | (31/D/PD/D) | Likely pathogenic | Likely pathogenic (PM1, PM2, PP2, PP3, PP4) |  |
| A1242 | 251 | *COL4A3* | c.2330G>A | p.G777D | Oka(2014)Pediatr Nephrol | NA | (29.4/D/PD/D) | NA | Likely pathogenic (PM1, PM2, PP2, PP3, PP4) |  |
| A1243 | 252 | *COL4A3* | c.469G>C | p.G157R | Xiong(2015)Science | 0 | (28.2/D/PD/D) | Pathogenic, Likely pathogenic | Likely pathogenic (PM1, PM2, PP2, PP4) |  |
| A1247 | 253 | *COL4A4* | c.1323_1340del | p.P444_L449del | Boye(1998)Am J Hum Genet | 8.9099×e-5 | NA | NA | Likely pathogenic (PM2, PM4, PP1, PP4) |  |
| A1248 | 254 | *COL4A3* | c.2746+2T>C |  |  | NA | NA | NA | Pathogenic (PVS1, PM2) |  |
| A1260 | 255 | *COL4A3* | c.4793T>G | p.L1598R | Oka(2014)Pediatr Nephrol | 0.00075754 | (28.4/D/PD/D) | Conflicting | Likely pathogenic (PM1, PM2, PP2, PP3) |  |
| A1261 | 256 | *COL4A4* | c.1891G>A | p.G631R |  | NA | (24.4/D/NA/D) | NA | Likely pathogenic (PM1, PM2, PP2, PP3, PP4) |  |
| A1270 | 257 | *COL4A3* | c.1229G>A | p.G410E | Oh(2021) Clin Genet | 2.7727×e-5 | (24.5/D/PD/D) | Pathogenic | Likely pathogenic (PM1, PM2, PP2, PP3) |  |
| A1275 | 258 | *COL4A3* | c.1994G>A | p.G665D | Horinouchi(2020)Kidney360 | NA | (24.7/D/PD/D) | NA | Likely pathogenic (PM1, PM2, PP1, PP2, PP3, PP4) |  |
| A1277 | 260 | *COL4A4* | c.1795G>C | p.G599R | Fallerini(2014)Clin Genet | NA | (24.2/D/PD/D) | NA | Likely pathogenic (PM1, PM2, PP2, PP3, PP4,) |  |
| A1287 | 261 | *COL4A4* | c.3973G>T | p.G1325* | Riedhmmer(2024)Clin Genet | NA | NA | Likely pathogenic | Pathogenic (PVS1, PM2) |  |
| A1292 | 262 | *COL4A4* | c.1442G>T | p.G481V | Bekheirnia(2021)Kidney360 | 0 | (25.7/D/PD/D) | NA | Likely pathogenic (PM1, PM2, PP1, PP2, PP3, PP4) |  |
| A1293 | 263 | *COL4A4* | c.1396G>A | p.G466R | Weber(2016) Pediatr Nephrol | 0 | (26.6/D/PD/D) | Pathogenic, Likely pathogenic | Likely pathogenic (PM1, PM2, PP1, PP2, PP3, PP4) |  |
| A1294 | 264 | *COL4A3* | c.1976G>A | p.G659Q |  | NA | (25.7/D/PD/D) | VUS | Likely pathogenic (PM1, PM2, PP1, PP2, PP3, PP4) |  |
| A1295 | 265 | *COL4A4* | c.4129C>T | p.R1377* | Boye(1998)Am J Hum Genet | 0 | NA | Pathogenic | Pathogenic (PVS1, PM2) |  |
| A1297 | 266 | *COL4A3* | c.1307G>C | p.G436A |  | 0.0001114 | (24.8/D/PD/D) | VUS | Likely pathogenic (PM1, PM2, PP1, PP2, PP3, PP4) |  |
| A1299 | 267 | *COL4A4* | c.1566G>A | p.W522* |  | NA | NA | NA | Pathogenic (PVS1, PM2) |  |
| A1300 | 268 | *COL4A4* | c.1535G>T | p.G512V |  | NA | (26.3/D/PD/D) | NA | Likely pathogenic (PM1, PM2, PP1, PP2, PP3, PP4) |  |
| NA: Not available, D: Disease causing or Damaging, PD: Possibly damaging, B: Benign | | | | | | | | | | |

Reference

1.	Marcocci E, Uliana V, Bruttini M, et al. Autosomal dominant Alport syndrome: molecular analysis of the COL4A4 gene and clinical outcome. *Nephrol Dial Transplant*. May 2009;24(5):1464-71. doi:10.1093/ndt/gfn681

2.	Fallerini C, Dosa L, Tita R, et al. Unbiased next generation sequencing analysis confirms the existence of autosomal dominant Alport syndrome in a relevant fraction of cases. *Clin Genet*. Sep 2014;86(3):252-7. doi:10.1111/cge.12258

3.	Morinière V, Dahan K, Hilbert P, et al. Improving mutation screening in familial hematuric nephropathies through next generation sequencing. *J Am Soc Nephrol*. Dec 2014;25(12):2740-51. doi:10.1681/asn.2013080912

4.	Oka M, Nozu K, Kaito H, et al. Natural history of genetically proven autosomal recessive Alport syndrome. *Pediatr Nephrol*. Sep 2014;29(9):1535-44. doi:10.1007/s00467-014-2797-4

5.	Mencarelli MA, Heidet L, Storey H, et al. Evidence of digenic inheritance in Alport syndrome. *J Med Genet*. Mar 2015;52(3):163-74. doi:10.1136/jmedgenet-2014-102822

6.	Xiong HY, Alipanahi B, Lee LJ, et al. RNA splicing. The human splicing code reveals new insights into the genetic determinants of disease. *Science*. Jan 9 2015;347(6218):1254806. doi:10.1126/science.1254806

7.	Kamiyoshi N, Nozu K, Fu XJ, et al. Genetic, Clinical, and Pathologic Backgrounds of Patients with Autosomal Dominant Alport Syndrome. *Clin J Am Soc Nephrol*. Aug 8 2016;11(8):1441-1449. doi:10.2215/cjn.01000116

8.	Weber S, Strasser K, Rath S, et al. Identification of 47 novel mutations in patients with Alport syndrome and thin basement membrane nephropathy. *Pediatr Nephrol*. Jun 2016;31(6):941-55. doi:10.1007/s00467-015-3302-4

9.	Papazachariou L, Papagregoriou G, Hadjipanagi D, et al. Frequent COL4 mutations in familial microhematuria accompanied by later-onset Alport nephropathy due to focal segmental glomerulosclerosis. *Clin Genet*. Nov 2017;92(5):517-527. doi:10.1111/cge.13077

10.	Bullich G, Domingo-Gallego A, Vargas I, et al. A kidney-disease gene panel allows a comprehensive genetic diagnosis of cystic and glomerular inherited kidney diseases. *Kidney Int*. Aug 2018;94(2):363-371. doi:10.1016/j.kint.2018.02.027

11.	Imafuku A, Nozu K, Sawa N, et al. Autosomal dominant form of type IV collagen nephropathy exists among patients with hereditary nephritis difficult to diagnose clinicopathologically. *Nephrology (Carlton)*. Oct 2018;23(10):940-947. doi:10.1111/nep.13115

12.	Sun Y, Man J, Wan Y, et al. Targeted next-generation sequencing as a comprehensive test for Mendelian diseases: a cohort diagnostic study. *Sci Rep*. Aug 3 2018;8(1):11646. doi:10.1038/s41598-018-30151-z

13.	Groopman EE, Marasa M, Cameron-Christie S, et al. Diagnostic Utility of Exome Sequencing for Kidney Disease. *N Engl J Med*. Jan 10 2019;380(2):142-151. doi:10.1056/NEJMoa1806891

14.	Rao J, Liu X, Mao J, et al. Genetic spectrum of renal disease for 1001 Chinese children based on a multicenter registration system. *Clin Genet*. Nov 2019;96(5):402-410. doi:10.1111/cge.13606

15.	Schapiro D, Daga A, Lawson JA, et al. Panel sequencing distinguishes monogenic forms of nephritis from nephrosis in children. *Nephrol Dial Transplant*. Mar 1 2019;34(3):474-485. doi:10.1093/ndt/gfy050

16.	Horinouchi T, Yamamura T, Nagano C, et al. Heterozygous Urinary Abnormality-Causing Variants of COL4A3 and COL4A4 Affect Severity of Autosomal Recessive Alport Syndrome. *Kidney360*. Sep 24 2020;1(9):936-942. doi:10.34067/kid.0000372019

17.	Bekheirnia N, Glinton KE, Rossetti L, et al. Clinical Utility of Genetic Testing in the Precision Diagnosis and Management of Pediatric Patients with Kidney and Urinary Tract Diseases. *Kidney360*. Jan 28 2021;2(1):90-104. doi:10.34067/kid.0002272020

18.	Oh J, Shin JI, Lee K, Lee C, Ko Y, Lee JS. Clinical application of a phenotype-based NGS panel for differential diagnosis of inherited kidney disease and beyond. *Clin Genet*. Feb 2021;99(2):236-249. doi:10.1111/cge.13869

19.	Zhang Y, Böckhaus J, Wang F, et al. Genotype-phenotype correlations and nephroprotective effects of RAAS inhibition in patients with autosomal recessive Alport syndrome. *Pediatr Nephrol*. Sep 2021;36(9):2719-2730. doi:10.1007/s00467-021-05040-9

20.	Al-Hamed MH, Hussein MH, Shah Y, et al. Exome sequencing unravels genetic variants associated with chronic kidney disease in Saudi Arabian patients. *Hum Mutat*. Dec 2022;43(12):e24-e37. doi:10.1002/humu.24480

21.	Bleyer AJ, Westemeyer M, Xie J, et al. Genetic Etiologies for Chronic Kidney Disease Revealed through Next-Generation Renal Gene Panel. *Am J Nephrol*. 2022;53(4):297-306. doi:10.1159/000522226

22.	Domingo-Gallego A, Pybus M, Bullich G, et al. Clinical utility of genetic testing in early-onset kidney disease: seven genes are the main players. *Nephrol Dial Transplant*. Mar 25 2022;37(4):687-696. doi:10.1093/ndt/gfab019

23.	Gao M, Yu F, Dong R, et al. Diagnostic application of exome sequencing in Chinese children with suspected inherited kidney diseases. *Front Genet*. 2022;13:933636. doi:10.3389/fgene.2022.933636

24.	García-Aznar JM, De la Higuera L, Besada Cerecedo L, et al. New Insights into Renal Failure in a Cohort of 317 Patients with Autosomal Dominant Forms of Alport Syndrome: Report of Two Novel Heterozygous Mutations in COL4A3. *J Clin Med*. Aug 19 2022;11(16)doi:10.3390/jcm11164883

25.	Ponleitner M, Allmer DM, Hecking M, et al. Phenotyping of a novel COL4A4 and novel GLA variant in a patient presenting with microhematuria and mildly impaired kidney function: a case report. *Front Genet*. 2023;14:1211858. doi:10.3389/fgene.2023.1211858

26.	Yuan X, Su Q, Wang H, et al. Genetic Variants of the COL4A3 , COL4A4 , and COL4A5 Genes Contribute to Thinned Glomerular Basement Membrane Lesions in Sporadic IgA Nephropathy Patients. *J Am Soc Nephrol*. Jan 1 2023;34(1):132-144. doi:10.1681/asn.2021111447

27.	Zhou L, Xi B, Xu Y, et al. Clinical, histological and molecular characteristics of Alport syndrome in Chinese children. *J Nephrol*. Jun 2023;36(5):1415-1423. doi:10.1007/s40620-023-01570-7

28.	Fujimaru T, Mori T, Chiga M, et al. Genetic Diagnosis of Adult Hemodialysis Patients With Unknown Etiology. *Kidney Int Rep*. Apr 2024;9(4):994-1004. doi:10.1016/j.ekir.2024.01.027

29.	Riedhammer KM, Simmendinger H, Tasic V, et al. Is there a dominant-negative effect in individuals with heterozygous disease-causing variants in COL4A3/COL4A4? *Clin Genet*. Apr 2024;105(4):406-414. doi:10.1111/cge.14471

Supplementary Table S3

| Patient | Family history of  haematuria or KF | | Age at  genetic diagnosis | sex | Haematuria | Proteinuria | KF | Hearing abnormality | Ocular abnormality | Kidney biopsy | Electron microscopic features of AS | Taking RAS inhibitors |
| --- | --- | --- | --- | --- | --- | --- | --- | --- | --- | --- | --- | --- |
| 72 | KF | | 14 | F | Yes | No | No | No | No | Yes | Yes | No |
| 86 | KF | | 34 | F | Yes | No | No | No | No | No | NA | Yes |
| 129 | KF | | 43 | M | Yes | Yes | No | No | No | Yes | Yes | No |
| 153 | KF | | 49 | F | Yes | Yes | No | No | No | Yes | Yes | Yes |
| 154 | haematuria | | 26 | F | Yes | Yes | No | No | No | Yes | Yes | No |
| 161 | none | | 28 | M | Yes | Yes | No | No | No | Yes | Yes | No |
| 168 | haematuria | | 19 | M | Yes | Yes | Yes | Yes | No | Yes | Yes | No |
| 175 | KF | | 31 | F | Yes | Yes | No | No | No | Yes | Yes | No |
| 198 | haematuria | | 36 | M | Yes | Yes | No | No | No | Yes | NA | Yes |
| 205 | KF | | 45 | M | Yes | Yes | No | No | No | Yes | Yes | No |
| 269 | KF | | 22 | M | Yes | No | No | Yes | No | Yes | Yes | Yes |
| 274 | none | | 6 | F | Yes | No | No | No | No | Yes | Yes | Yes |
| 280 | haematuria | | 67 | F | Yes | Yes | No | No | No | Yes | Yes | Yes |
| 285 | KF | | 24 | F | Yes | No | No | No | No | Yes | Yes | No |
| 291 | none | | 30 | F | Yes | Yes | No | No | No | Yes | Yes | Yes |
| 297 | haematuria | | 11 | F | Yes | Yes | No | No | No | Yes | Yes | Yes |
| 304 | haematuria | | 47 | F | Yes | Yes | No | Yes | No | Yes | Yes | No |
| 305 | KF | | 69 | F | Yes | Yes | No | Yes | No | Yes | Yes | Yes |
| 315 | haematuria | | 51 | M | Yes | Yes | No | No | No | Yes | Yes | Yes |
| 319 | KF | | 49 | F | Yes | Yes | No | No | No | Yes | Yes | Yes |
| 320 | haematuria | | 50 | M | Yes | Yes | Yes | Yes | No | Yes | Yes | Yes |
| 330 | haematuria | | 56 | F | Yes | Yes | No | No | No | Yes | Yes | Yes |
| 331 | KF | | 29 | F | Yes | Yes | No | No | No | Yes | Yes | Yes |
| 332 | KF | | 30 | M | Yes | Yes | Yes | No | No | Yes | Yes | Yes |
| 335 | KF | | 54 | M | Yes | Yes | No | No | No | Yes | Yes | Yes |
| 336 | KF | | 32 | M | Yes | Yes | No | No | No | Yes | Yes | Yes |
| 342 | KF | | 60 | F | Yes | Yes | No | No | No | Yes | Yes | Yes |
| 343 | none | | 4 | F | Yes | No | No | No | No | Yes | Yes | No |
| 350 | KF | | 6 | F | Yes | No | No | No | No | No | NA | No |
| 351 | KF | | 9 | M | Yes | Yes | No | No | No | Yes | Yes | Yes |
| 360 | KF | | 45 | F | Yes | Yes | No | No | No | Yes | Yes | Yes |
| 361 | haematuria | | 29 | F | Yes | Yes | No | No | No | Yes | Yes | No |
| 363 | haematuria | | 49 | M | Yes | Yes | Yes | Yes | No | Yes | NA | No |
| 367 | KF | | 47 | F | Yes | Yes | No | No | No | Yes | No | Yes |
| 369 | none | | 29 | F | Yes | Yes | No | Yes | No | Yes | Yes | No |
| 376 | haematuria | | 42 | M | Yes | Yes | No | No | No | Yes | Yes | No |
| 387 | haematuria | | 38 | F | Yes | Yes | No | No | No | Yes | Yes | No |
| 391 | haematuria | | 71 | F | Yes | Yes | No | No | No | Yes | Yes | Yes |
| 394 | haematuria | | 45 | M | Yes | Yes | Yes | Yes | No | Yes | NA | Yes |
| 407 | haematuria | | 19 | F | Yes | No | No | No | No | Yes | Yes | No |
| 408 | KF | | 44 | M | Yes | Yes | No | No | No | Yes | Yes | No |
| 418 | haematuria | | 43 | F | Yes | Yes | No | No | No | Yes | Yes | Yes |
| 420 | KF | | 19 | F | Yes | No | No | No | No | No | NA | No |
| 423 | haematuria | | 32 | F | Yes | Yes | No | No | No | Yes | Yes | No |
| 425 | none | | 27 | F | Yes | Yes | No | No | No | Yes | Yes | No |
| 433 | haematuria | | 36 | M | Yes | Yes | No | No | No | Yes | Yes | Yes |
| 434 | haematuria | | 10 | F | Yes | Yes | No | No | No | Yes | Yes | Yes |
| 437 | haematuria | | 41 | F | Yes | Yes | No | No | No | Yes | Yes | No |
| 439 | KF | | 10 | F | Yes | No | No | No | No | No | NA | No |
| 441 | none | | 39 | M | Yes | Yes | No | No | No | Yes | Yes | Yes |
| 444 | none | | 7 | M | Yes | Yes | No | No | No | Yes | Yes | Yes |
| 445 | none | | 47 | F | Yes | Yes | No | No | No | Yes | NA | Yes |
| 451 | KF | | 25 | M | Yes | Yes | No | No | No | Yes | Yes | Yes |
| 453 | none | | 46 | F | Yes | Yes | No | No | No | Yes | Yes | No |
| 457 | none | | 13 | F | Yes | Yes | Yes | Yes | No | Yes | Yes | No |
| 461 | none | | 41 | F | Yes | Yes | No | No | No | Yes | Yes | No |
| 464 | haematuria | | 18 | M | Yes | No | No | No | No | Yes | Yes | No |
| 466 | haematuria | | 4 | F | Yes | No | No | No | No | No | NA | No |
| 474 | KF | | 42 | F | Yes | No | No | No | No | Yes | NA | Yes |
| 477 | haematuria | | 6 | F | Yes | Yes | No | No | No | Yes | Yes | No |
| 495 | KF | | 51 | M | Yes | Yes | No | No | No | Yes | Yes | Yes |
| 504 | KF | | 59 | F | Yes | Yes | No | Yes | No | Yes | Yes | Yes |
| 512 | KF | | 49 | F | Yes | Yes | No | No | No | Yes | Yes | Yes |
| 518 | haematuria | | 21 | F | Yes | Yes | No | No | No | Yes | Yes | No |
| 522 | none | | 46 | M | Yes | No | No | No | No | Yes | Yes | Yes |
| 523 | none | | 43 | F | Yes | Yes | No | No | No | Yes | Yes | Yes |
| 531 | KF | | 29 | F | Yes | No | No | No | No | No | NA | No |
| 538 | haematuria | | 12 | M | Yes | Yes | No | No | No | Yes | Yes | Yes |
| 542 | KF | | 41 | M | Yes | No | No | No | No | Yes | Yes | No |
| 543 | haematuria | | 19 | M | Yes | No | No | No | No | No | NA | No |
| 544 | none | | 51 | M | Yes | Yes | No | No | No | Yes | Yes | No |
| 547 | KF | | 14 | M | Yes | No | No | No | No | No | NA | No |
| 551 | KF | | 51 | F | Yes | Yes | No | No | No | Yes | Yes | Yes |
| 563 | KF | | 22 | M | Yes | Yes | No | No | No | Yes | Yes | No |
| 565 | haematuria | | 43 | M | Yes | Yes | No | No | No | Yes | Yes | Yes |
| 568 | KF | | 26 | F | Yes | No | No | No | No | Yes | Yes | No |
| 580 | none | | 25 | F | Yes | Yes | No | No | No | NA | NA | No |
| 586 | KF | | 62 | M | Yes | Yes | No | No | No | Yes | NA | No |
| 590 | KF | | 44 | F | Yes | Yes | No | No | No | No | NA | No |
| 591 | haematuria | | 46 | F | Yes | Yes | No | No | No | Yes | Yes | No |
| 600 | haematuria | | 50 | F | Yes | Yes | No | No | No | Yes | Yes | Yes |
| 607 | KF | | 46 | F | Yes | Yes | No | No | No | Yes | Yes | Yes |
| 610 | haematuria | | 5 | F | Yes | Yes | No | No | No | No | NA | No |
| 613 | haematuria | | 30 | F | Yes | Yes | No | No | No | Yes | Yes | Yes |
| 619 | KF | | 43 | F | Yes | Yes | No | No | No | Yes | Yes | No |
| 627 | haematuria | | 5 | M | Yes | Yes | No | No | No | Yes | Yes | No |
| 630 | none | | 41 | F | Yes | Yes | No | No | No | Yes | Yes | No |
| 631 | haematuria | | 48 | M | Yes | Yes | No | No | No | Yes | Yes | Yes |
| 641 | haematuria | | 44 | F | Yes | Yes | No | No | No | Yes | Yes | Yes |
| 642 | haematuria | | 63 | F | Yes | Yes | No | No | No | Yes | Yes | No |
| 645 | KF | | 39 | M | Yes | Yes | No | No | No | Yes | NA | Yes |
| 648 | KF | | 33 | M | Yes | Yes | No | Yes | No | Yes | Yes | Yes |
| 655 | haematuria | | 7 | F | Yes | Yes | No | No | No | No | NA | No |
| 661 | none | | 51 | F | Yes | Yes | No | No | No | Yes | Yes | Yes |
| 668 | KF | | 62 | F | Yes | Yes | No | No | No | No | NA | Yes |
| 675 | haematuria | | 33 | F | Yes | Yes | No | No | No | Yes | Yes | Yes |
| 683 | none | | 59 | F | Yes | Yes | No | No | No | Yes | Yes | No |
| 693 | none | | 40 | M | Yes | Yes | No | No | No | Yes | Yes | No |
| 695 | haematuria | | 42 | F | Yes | Yes | No | No | No | Yes | NA | Yes |
| 707 | none | | 57 | F | Yes | Yes | No | Yes | Yes | Yes | Yes | Yes |
| 723 | KF | | 25 | F | Yes | Yes | No | No | No | Yes | Yes | No |
| 730 | haematuria | | 39 | M | Yes | Yes | No | No | No | Yes | Yes | Yes |
| 734 | haematuria | | 50 | M | Yes | Yes | No | No | No | Yes | Yes | Yes |
| 742 | haematuria | | 54 | M | Yes | Yes | No | No | No | Yes | Yes | Yes |
| 745 | haematuria | | 57 | F | Yes | Yes | No | No | No | NA | NA | No |
| 746 | haematuria | | 40 | F | Yes | Yes | No | No | No | Yes | No | No |
| 752 | haematuria | | 35 | F | Yes | Yes | No | Yes | No | Yes | No | Yes |
| 756 | none | | 15 | F | Yes | Yes | No | No | No | Yes | Yes | Yes |
| 762 | none | | 38 | M | Yes | Yes | No | No | No | Yes | NA | Yes |
| 772 | haematuria | | 58 | F | Yes | Yes | No | No | No | Yes | Yes | No |
| 776 | haematuria | | 35 | M | Yes | Yes | No | No | No | NA | NA | Yes |
| 777 | KF | | 71 | F | Yes | Yes | No | No | No | Yes | No | Yes |
| 778 | haematuria | | 58 | F | Yes | Yes | No | No | No | Yes | Yes | No |
| 784 | haematuria | | 42 | F | Yes | Yes | No | No | No | Yes | Yes | No |
| 788 | haematuria | | 58 | F | Yes | Yes | No | No | No | Yes | Yes | No |
| 791 | KF | | 28 | M | Yes | Yes | No | No | No | Yes | Yes | No |
| 793 | haematuria | | 50 | M | Yes | Yes | No | No | No | Yes | Yes | Yes |
| 798 | KF | | 52 | F | Yes | NA | No | No | No | No | NA | No |
| 799 | KF | | 54 | M | Yes | Yes | No | Yes | No | Yes | Yes | Yes |
| 804 | haematuria | | 1 | M | Yes | Yes | No | No | No | No | NA | No |
| 808 | KF | | 60 | F | Yes | Yes | No | No | Yes | Yes | NA | Yes |
| 809 | haematuria | | 36 | F | Yes | Yes | No | No | No | Yes | Yes | Yes |
| 819 | KF | | 18 | M | Yes | No | No | No | No | Yes | Yes | No |
| 826 | haematuria | | 50 | M | Yes | Yes | Yes | No | No | No | NA | No |
| 833 | none | | 56 | F | Yes | Yes | No | No | No | Yes | NA | No |
| 836 | none | | 35 | F | Yes | No | No | No | No | Yes | Yes | No |
| 838 | KF | | 33 | F | Yes | Yes | No | No | No | Yes | Yes | No |
| 849 | haematuria | | 49 | F | Yes | Yes | No | No | No | No | NA | NA |
| 851 | KF | | 55 | F | Yes | Yes | No | No | No | Yes | Yes | NA |
| 867 | KF | | 49 | M | Yes | No | No | No | No | NA | NA | Yes |
| 871 | KF | | 47 | F | Yes | Yes | No | No | No | Yes | No | Yes |
| 872 | KF | | 75 | F | Yes | Yes | No | No | No | No | NA | Yes |
| 874 | KF | | 49 | F | Yes | No | No | Yes | No | Yes | Yes | Yes |
| 875 | none | | 37 | M | Yes | No | No | No | No | Yes | NA | NA |
| 877 | haematuria | | 33 | M | Yes | Yes | Yes | No | No | Yes | Yes | Yes |
| 879 | none | | 48 | F | Yes | Yes | No | No | No | Yes | Yes | NA |
| 883 | none | | 37 | F | Yes | Yes | No | No | No | Yes | Yes | Yes |
| 885 | KF | | 50 | M | Yes | Yes | No | Yes | No | Yes | Yes | Yes |
| 889 | haematuria | | 56 | F | Yes | Yes | No | No | No | Yes | Yes | Yes |
| 902 | haematuria | | 11 | M | Yes | No | No | No | No | No | NA | NA |
| 916 | haematuria | | 8 | F | Yes | No | No | No | No | No | NA | NA |
| 918 | none | | 16 | F | Yes | Yes | No | No | No | Yes | Yes | Yes |
| 920 | haematuria | | 63 | M | Yes | Yes | No | Yes | No | Yes | Yes | NA |
| 922 | KF | | 45 | M | Yes | Yes | No | No | No | Yes | Yes | NA |
| 923 | KF | | 59 | M | Yes | Yes | No | No | No | Yes | Yes | NA |
| 927 | haematuria | | 49 | F | Yes | Yes | No | Yes | No | Yes | Yes | NA |
| 929 | haematuria | | 47 | F | Yes | Yes | No | No | No | Yes | Yes | NA |
| 931 | KF | | 15 | M | Yes | No | No | No | No | No | NA | NA |
| 934 | none | | 48 | F | Yes | Yes | No | No | No | Yes | Yes | Yes |
| 937 | KF | | 28 | F | Yes | No | No | No | No | No | NA | NA |
| 940 | haematuria | | 69 | F | Yes | Yes | No | Yes | No | Yes | NA | NA |
| 944 | haematuria | | 64 | F | Yes | Yes | No | No | No | Yes | Yes | Yes |
| 949 | haematuria | | 50 | M | Yes | Yes | No | No | No | Yes | Yes | Yes |
| 956 | KF | | 63 | F | Yes | Yes | No | No | No | Yes | Yes | NA |
| 957 | none | | 60 | F | Yes | No | No | No | No | Yes | Yes | NA |
| 959 | haematuria | | 37 | M | Yes | No | No | No | No | No | NA | NA |
| 961 | haematuria | | 58 | F | Yes | Yes | No | No | No | Yes | Yes | Yes |
| 966 | haematuria | | 64 | F | Yes | Yes | No | No | No | Yes | No | Yes |
| 970 | haematuria | | 8 | M | Yes | Yes | No | No | No | No | NA | NA |
| 971 | KF | | 38 | M | Yes | Yes | No | No | No | Yes | NA | Yes |
| 973 | KF | | 2 | F | Yes | Yes | No | No | No | Yes | Yes | NA |
| 975 | haematuria | | 42 | F | Yes | Yes | No | No | No | Yes | Yes | Yes |
| 976 | KF | | 15 | M | Yes | No | No | No | No | No | NA | NA |
| 978 | KF | | 54 | F | Yes | Yes | No | No | No | Yes | Yes | NA |
| 979 | KF | | 42 | M | Yes | Yes | No | No | No | Yes | Yes | Yes |
| 983 | haematuria | | 68 | M | Yes | Yes | No | No | No | Yes | NA | Yes |
| 985 | haematuria | | 36 | F | Yes | Yes | No | No | No | Yes | Yes | NA |
| 986 | KF | | 57 | M | Yes | Yes | No | No | No | Yes | NA | Yes |
| 988 | KF | | 49 | F | Yes | Yes | No | No | No | Yes | Yes | NA |
| 994 | none | | 15 | M | Yes | Yes | No | No | No | Yes | Yes | Yes |
| 999 | haematuria | | 58 | F | Yes | Yes | No | No | No | Yes | Yes | Yes |
| 1002 | haematuria | | 4 | F | Yes | Yes | No | No | No | Yes | Yes | NA |
| 1011 | KF | | 43 | F | Yes | Yes | No | No | No | Yes | Yes | Yes |
| 1012 | none | | 65 | F | Yes | Yes | No | No | No | Yes | Yes | Yes |
| 1013 | haematuria | | 56 | F | Yes | Yes | No | No | No | Yes | NA | NA |
| 1016 | haematuria | | 69 | F | Yes | Yes | No | No | No | No | NA | Yes |
| 1028 | none | | 65 | F | Yes | Yes | No | No | No | Yes | Yes | NA |
| 1029 | KF | | 43 | F | Yes | Yes | No | No | No | No | NA | NA |
| 1030 | haematuria | | 11 | M | Yes | Yes | No | No | No | Yes | Yes | Yes |
| 1035 | haematuria | | 15 | M | Yes | Yes | No | No | No | No | NA | NA |
| 1041 | haematuria | | 28 | F | Yes | Yes | No | No | No | Yes | Yes | NA |
| 1044 | haematuria | | 32 | F | Yes | No | No | No | No | NA | NA | NA |
| 1046 | haematuria | | 39 | F | Yes | Yes | No | No | No | Yes | Yes | NA |
| 1048 | KF | | 28 | F | Yes | Yes | No | No | No | No | NA | NA |
| 1049 | haematuria | | 37 | F | Yes | Yes | No | No | No | Yes | Yes | Yes |
| 1053 | haematuria | | 51 | F | Yes | Yes | No | No | No | Yes | Yes | NA |
| 1055 | haematuria | | 15 | M | Yes | Yes | No | No | No | Yes | Yes | Yes |
| 1057 | haematuria | | 37 | F | Yes | No | No | No | No | Yes | Yes | NA |
| 1058 | haematuria | | 70 | M | Yes | Yes | No | Yes | No | Yes | Yes | Yes |
| 1067 | KF | | 37 | F | Yes | Yes | No | No | No | Yes | Yes | Yes |
| 1069 | KF | | 40 | F | Yes | Yes | No | No | No | Yes | Yes | NA |
| 1072 | KF | | 3 | F | Yes | Yes | No | No | No | No | NA | NA |
| 1078 | haematuria | | 65 | F | Yes | No | No | No | No | Yes | No | NA |
| 1079 | KF | | 63 | M | Yes | Yes | No | Yes | No | Yes | Yes | Yes |
| 1080 | haematuria | | 56 | F | Yes | Yes | No | No | No | Yes | Yes | Yes |
| 1081 | none | | 23 | F | Yes | Yes | No | No | No | Yes | Yes | Yes |
| 1087 | none | | 56 | F | Yes | Yes | No | No | No | Yes | Yes | Yes |
| 1094 | haematuria | | 29 | F | Yes | Yes | No | No | No | Yes | NA | NA |
| 1096 | haematuria | | 35 | F | Yes | Yes | No | No | No | Yes | Yes | Yes |
| 1097 | haematuria | | 34 | F | Yes | Yes | No | No | No | Yes | Yes | Yes |
| 1104 | haematuria | | 46 | M | Yes | No | No | No | No | Yes | No | NA |
| 1108 | haematuria | | 21 | F | Yes | No | No | No | No | No | NA | NA |
| 1110 | none | | 8 | F | Yes | Yes | No | No | No | Yes | Yes | NA |
| 1111 | KF | | 68 | F | Yes | Yes | No | No | No | Yes | NA | Yes |
| 1115 | haematuria | | 11 | M | Yes | No | No | No | No | Yes | Yes | NA |
| 1120 | none | | 44 | M | Yes | No | No | No | No | Yes | Yes | NA |
| 1121 | KF | | 32 | F | Yes | Yes | No | No | No | Yes | Yes | Yes |
| 1123 | none | | 23 | M | Yes | No | No | No | No | Yes | No | NA |
| 1128 | haematuria | | 76 | M | Yes | Yes | No | No | No | No | NA | Yes |
| 1129 | haematuria | | 35 | F | Yes | Yes | No | No | No | Yes | Yes | Yes |
| 1130 | haematuria | | 35 | M | Yes | No | No | No | No | No | NA | Yes |
| 1131 | KF | | 67 | M | Yes | Yes | No | No | No | No | NA | Yes |
| 1135 | haematuria | | 42 | F | Yes | Yes | No | No | No | Yes | Yes | Yes |
| 1137 | KF | | 48 | M | Yes | Yes | Yes | No | No | Yes | NA | Yes |
| 1139 | haematuria | | 61 | F | Yes | Yes | No | No | No | Yes | Yes | Yes |
| 1143 | haematuria | | 15 | F | Yes | Yes | No | No | No | Yes | Yes | NA |
| 1151 | haematuria | | 7 | M | Yes | Yes | No | No | No | Yes | Yes | Yes |
| 1157 | KF | | 42 | F | Yes | No | No | No | No | No | NA | NA |
| 1158 | haematuria | | 46 | M | Yes | Yes | No | No | No | Yes | Yes | Yes |
| 1159 | KF | | 57 | F | Yes | Yes | No | No | No | Yes | Yes | Yes |
| 1160 | KF | | 31 | F | Yes | Yes | No | No | No | Yes | NA | NA |
| 1161 | haematuria | | 11 | M | Yes | No | No | No | No | No | NA | NA |
| 1171 | haematuria | | 16 | F | Yes | No | No | No | No | No | NA | NA |
| 1172 | KF | | 43 | M | Yes | Yes | No | No | No | Yes | Yes | Yes |
| 1173 | none | | 5 | F | Yes | Yes | No | No | No | Yes | Yes | NA |
| 1177 | KF | | 63 | F | Yes | Yes | No | Yes | No | Yes | Yes | NA |
| 1185 | KF | | 55 | M | Yes | Yes | No | No | No | Yes | Yes | Yes |
| 1187 | haematuria | | 48 | M | Yes | Yes | No | No | No | No | NA | Yes |
| 1188 | haematuria | | 65 | F | Yes | Yes | No | Yes | No | Yes | Yes | Yes |
| 1189 | none | | 73 | F | Yes | Yes | No | No | No | Yes | NA | NA |
| 1191 | haematuria | | 37 | M | Yes | Yes | No | No | No | Yes | No | Yes |
| 1192 | haematuria | | 43 | F | Yes | Yes | No | No | No | Yes | Yes | Yes |
| 1195 | haematuria | | 49 | M | Yes | Yes | No | No | No | Yes | Yes | Yes |
| 1198 | haematuria | | 6 | M | Yes | No | No | No | No | Yes | Yes | NA |
| 1201 | haematuria | | 46 | F | Yes | Yes | No | No | No | Yes | Yes | Yes |
| 1203 | haematuria | | 27 | F | Yes | Yes | No | No | No | Yes | Yes | NA |
| 1210 | haematuria | | 75 | F | Yes | Yes | No | No | No | Yes | NA | Yes |
| 1211 | haematuria | | 9 | F | Yes | No | No | No | No | No | NA | NA |
| 1212 | haematuria | | 8 | M | Yes | No | No | No | No | No | NA | NA |
| 1216 | haematuria | | 14 | F | Yes | Yes | No | No | No | Yes | Yes | Yes |
| 1067a | KF | | 67 | F | Yes | Yes | No | No | No | Yes | NA | Yes |
| 1067b | KF | | 12 | F | Yes | No | No | No | No | No | NA | No |
| 1096a | haematuria | | 70 | F | Yes | Yes | No | No | No | No | NA | Yes |
| 1097a | haematuria | | 4 | F | Yes | No | No | No | No | No | NA | No |
| 1111a | KF | | 36 | M | Yes | No | No | No | No | No | NA | NA |
| 1115a | haematuria | | 48 | M | Yes | Yes | No | Yes | No | Yes | Yes | NA |
| 1115b | haematuria | | 8 | M | Yes | No | No | No | No | Yes | Yes | NA |
| 1121a | KF | | 62 | M | Yes | Yes | Yes | No | No | No | NA | Yes |
| 1128a | haematuria | | 45 | F | Yes | No | No | No | No | No | NA | NA |
| 1128b | haematuria | | 41 | F | Yes | No | No | No | No | No | NA | NA |
| 1130b | haematuria | | 59 | F | Yes | Yes | No | No | No | No | NA | NA |
| 1130c | haematuria | | 34 | M | Yes | No | No | No | No | No | NA | No |
| 1137a | KF | | 13 | F | Yes | No | No | No | No | No | NA | NA |
| 1157a | KF | | 37 | F | Yes | No | No | No | No | No | NA | NA |
| 153e | KF | | 83 | M | Yes | Yes | Yes | No | No | No | NA | No |
| 304a | haematuria | | 20 | F | Yes | No | No | No | No | Yes | NA | No |
| 304b | haematuria | | 16 | F | Yes | No | No | No | No | No | NA | No |
| 304c | haematuria | | 73 | F | Yes | No | No | No | No | No | NA | No |
| 342A | KF | | 36 | M | Yes | Yes | No | No | No | No | NA | No |
| 351A | KF | | 11 | F | Yes | Yes | No | No | No | No | NA | No |
| 351B | KF | | 6 | F | Yes | No | No | No | No | No | NA | No |
| 394 b | haematuria | | 71 | F | Yes | No | No | No | No | No | NA | No |
| 568A | KF | | 58 | F | Yes | No | Yes | No | No | Yes | Yes | No |
| 695A | haematuria | | 42 | F | Yes | Yes | No | No | No | Yes | Yes | Yes |
| 86a | KF | | 14 | F | Yes | No | No | No | No | No | NA | No |
| 872a | KF | | 50 | M | Yes | Yes | Yes | No | No | No | NA | NA |
| 916a | haematuria | | 36 | F | Yes | Yes | No | No | No | Yes | NA | No |
| 916b | haematuria | | 6 | F | Yes | Yes | No | No | No | Yes | NA | No |
| 922a | KF | | 69 | M | Yes | No | Yes | No | No | No | NA | NA |
| 937a | KF | | 57 | F | Yes | Yes | No | No | No | No | NA | NA |
| 978a | KF | | 27 | M | Yes | No | No | No | No | No | NA | NA |
| 986a | KF | | 28 | M | Yes | No | No | No | No | No | NA | NA |
| 986b | KF | | 24 | M | Yes | No | No | No | No | No | NA | NA |
| A1218 | none | | 59 | F | Yes | Yes | No | Yes | No | Yes | Yes | Yes |
| A1219 | haematuria | | 48 | F | Yes | Yes | No | Yes | No | Yes | Yes | Yes |
| A1232 | haematuria | | 40 | F | Yes | Yes | No | No | No | Yes | Yes | No |
| A1234 | haematuria | | 39 | F | Yes | Yes | No | No | No | No | NA | No |
| A1236 | KF | | 58 | F | Yes | Yes | No | No | No | Yes | Yes | No |
| A1238 | KF | | 54 | F | Yes | Yes | No | No | No | Yes | Yes | Yes |
| A1239 | KF | | 56 | M | Yes | Yes | No | No | No | Yes | Yes | Yes |
| A1240 | haematuria | | 54 | F | Yes | Yes | No | No | No | Yes | Yes | Yes |
| A1242 | KF | | 64 | F | Yes | Yes | No | No | No | No | NA | Yes |
| A1243 | haematuria | | 40 | F | Yes | Yes | No | No | No | No | NA | Yes |
| A1247 | haematuria | | 59 | F | Yes | No | No | No | No | Yes | Yes | No |
| A1248 | KF | | 34 | M | Yes | Yes | No | No | No | No | NA | Yes |
| A1260 | none | | 37 | M | Yes | No | No | Yes | No | NA | Yes | No |
| A1261 | KF | | 31 | F | Yes | Yes | No | No | No | Yes | Yes | Yes |
| A1270 | none | | 48 | F | Yes | Yes | No | No | No | Yes | Yes | Yes |
| A1275 | KF | | 48 | F | Yes | No | No | No | No | Yes | NA | No |
| A1275a | KF | | 72 | F | Yes | No | No | No | No | Yes | Yes | Yes |
| A1277 | haematuria | | 40 | F | Yes | Yes | No | No | No | Yes | Yes | No |
| A1287 | haematuria | | 50 | F | Yes | Yes | No | No | No | Yes | Yes | Yes |
| A1292 | KF | | 48 | M | Yes | Yes | No | No | No | Yes | NA | No |
| A1293 | haematuria | | 20 | M | Yes | No | No | No | No | Yes | Yes | No |
| A1294 | KF | | 9 | M | Yes | No | No | No | No | No | NA | No |
| A1295 | none | | 45 | F | Yes | Yes | No | No | No | Yes | Yes | Yes |
| A1297 | KF | | 22 | M | Yes | No | No | No | No | Yes | NA | No |
| A1299 | KF | | 26 | F | Yes | Yes | No | No | No | Yes | Yes | No |
| A1300 | haematuria | | 68 | F | Yes | Yes | No | No | No | Yes | Yes | Yes |
|  | |  |  |  |  |  |  |  |  |  |  |  |

Supplementary Table S4

| ID | Sex | Gene | Variant | RAS-I therapy | Comorbidities | | | | | | | |
| --- | --- | --- | --- | --- | --- | --- | --- | --- | --- | --- | --- | --- |
|  |  |  |  |  | hypertension | obesity | diabetes mellitus | hyperuricaemia | dyslipidaemia | Kidney disease other than AS | Other diseases | |
| 1137 | M | *COL4A3* | Missense | Yes | No | Yes | No | Yes | Yes | No | No |  |
| 877 | M | *COL4A3* | Missense | Yes | No | No | No | Yes | Yes | No | No |  |
| 826 | M | *COL4A4* | Missense | No | No | Yes | No | No | Yes | No | No |  |
| 320 | M | *COL4A3* | Missense | Yes | No | No | No | No | No | No | No |  |
| 332 | M | *COL4A4* | Missense | Yes | No | Yes | No | Yes | Yes | No | No |  |
| 363 | M | *COL4A3* | Splice site | No | Yes | No | No | No | No | No | No |  |
| 394 | M | *COL4A4* | Missense | Yes | No | No | No | No | Yes | No | No |  |
| 168 | M | *COL4A3* | Missense | NA | No | No | NA | NA | NA | NA | NA |  |
| 457 | F | *COL4A3* | Indel | Yes | No | No | No | No | No | No | No |  |
| 1121a | M | *COL4A4* | Indel | Yes | No | Yes | No | No | No | No | No |  |
| 153e | F | *COL4A4* | Missense | NA | Yes | No | No | Yes | No | No | aortic aneurysm, atrial fibrillation |  |
| 568A | F | *COL4A4* | Missense | NA | NA | Yes | NA | NA | NA | NA | NA |  |
| 872a | M | *COL4A4* | Missense | NA | No | Yes | No | No | No | No | No |  |
| 922a | M | *COL4A3* | Missense | NA | Yes | No | No | NA | NA | No | Atrial fibrillation, hyperthyroidism, alcohol-related chronic hepatitis |  |

RAS-I: Renin-angiotensin system inhibitor, NA: not available, F: female, M: male, AS: Alport syndrome

Supplementary Table S5

| ID | Age at genetic diagnosis | Sex | Gene | Variant | Age at appearing  Haematuria | Age at appearing Proteinuria | Cr-eGFR at diagnosis | RAS-I  therapy | Gross haematuria | obesity | Kidney disease other than AS | Other  disease | EM  findings |
| --- | --- | --- | --- | --- | --- | --- | --- | --- | --- | --- | --- | --- | --- |
| 940 | 69 | F | *COL4A3* | Missense | 40 | 40 | 38 | No | No | No | No |  | No KBx |
| 923 | 59 | M | *COL4A3* | Missense | 10 | 56 | 39 | No | No | No | No | HT, HU | TBM |
| 1130 | 59 | F | *COL4A4* | Missense | 46 | 46 | 48 | No | Yes | No | No |  | No KBx |
| 1130C | 33 | M | *COL4A4* | Missense | NA | Non | 59 | Yes | No | No | No |  | No KBx |
| 1057 | 37 | F | *COL4A4* | Missense | 34 | Non | 49 | No | No | No | No | HT, Hypothyroidism | TBM |
| 988 | 49 | F | *COL4A4* | Missense | 4 | 46 | 51 | No | Yes | Yes | No |  | BWC |
| 867 | 49 | M | *COL4A3* | Missense | 10 | 10 | 54 | Yes | Yes | No | No |  | NA |
| 957 | 60 | F | *COL4A4* | Missense | 22 | Non | 58 | No | No | No | No |  | TBM |
| 522 | 46 | M | *COL4A4* | Missense | 3 | Non | 48 | Yes | No | No | No | HT | TBM |
| 568 | 26 | F | *COL4A4* | Missense | 3 | Non | 48 | No | No | No | No |  | BWC |
| 568A | 58 | F | *COL4A4* | Missense | 16 | Non | KRT | No | No | Yes | No |  | TBM |
| 495 | 51 | M | *COL4A4* | Splice site | 19 | Non | 35 | Yes | No | Yes | No |  | BWC |
| 1078 | 65 | F | *COL4A3* | Missense | 45 | Non | 49 | No | No | No | No |  | TBM |
| 1275A | 72 | M | *COL4A3* | Missense | 14 | Non | 19 | Yes | No | No | No |  | TBM |
| 1275 | 48 | F | *COL4A3* | Missense | Childhood | Non | 36 | No | No | No | No |  | No KBx |

Cr-eGFR: Creatinine estimated glomerular filtration rate, RAS-I: Renin-angiotensin system inhibitor, EM: Electron microscopy, NA: not available, F: female, M: male,
 KBx：Kidney biopsy, TBM：Thin basement membrane, BWC：basket-weave change, HT: Hypertension, HU: Hyperuricemia, KRT: Kidney replacement therapy

Supplementary Figure S1
